# An isoform of AIF1 involved in breast cancer

**DOI:** 10.1186/s12935-018-0663-3

**Published:** 2018-10-22

**Authors:** Ferial Amira Slim, Geneviève Ouellette, Kaoutar Ennour-Idrissi, Simon Jacob, Caroline Diorio, Francine Durocher

**Affiliations:** 1 CHU de Québec Research Centre and Cancer Research Centre-Laval University, 2705 Laurier Boulevard, Quebec City, QC G1V 4G2 Canada; 2 Department of Molecular Medicine, Faculty of Medicine, Laval University, Quebec City, QC Canada; 3 Department of Social and Preventive Medicine, Laval University, Quebec City, QC Canada; 4 Pathology Laboratory, St-Sacrement Hospital, CHU de Quebec-Laval University, Quebec City, QC Canada

**Keywords:** Breast cancer, AIF1, Isoforms, Breast tumors, Tumor microenvironment, Inflammation

## Abstract

**Background:**

Inflammation is a major player in breast cancer (BC) progression. Allograft-inflammatory factor-1 (AIF1) is a crucial mediator in the inflammatory response. AIF1 reportedly plays a role in BC, but the mechanism remains to be elucidated. We identified two AIF1 isoforms, AIF1v1 and AIF1v3, which were differentially expressed between affected and unaffected sisters from families with high risk of BC with no deleterious BRCA1/BRCA2 mutations (BRCAX). We investigated potential functions of AIFv1/v3 in BC of varying severity and breast adipose tissue by evaluating their expression, and association with metabolic and clinical parameters of BC patients.

**Methods:**

AIF1v1/v3 expression was determined in BC tissues and cell lines using quantitative real-time PCR. Potential roles and mechanisms were examined in the microenvironment (fibroblasts, adipose tissue, monocytes and macrophages), inflammatory response (cell reaction in BC subgroups), and metabolism [treatment with docosahexaenoic acid (DHA)]. Association of AIF1 transcript expression with clinical factors was determined by Spearman’s rank correlation. Bioinformatics analyses were performed to characterize transcripts.

**Results:**

*AIF1v1/v3* were mostly expressed in the less severe BC samples, and their expression appeared to originate from the tumor microenvironment. *AIF1* isoforms had different expression rates and sources in breast adipose tissue; lymphocytes mostly expressed *AIF1v1* while activated macrophages mainly expressed *AIF1v3*. Bioinformatics analysis revealed major structural differences suggesting distinct functions in BC progression. Lymphocytes were the most infiltrating cells in breast tumors and their number correlated with *AIF1v1* adipose expression. Furthermore, DHA supplementation significantly lowered the expression of *AIF1* isoforms in BRCAX cell lines. Finally, the expression of *AIF1* isoforms in BC and breast adipose tissue correlated with clinical parameters of BC patients.

**Conclusions:**

Results strongly suggest that AIF1v1 as much as AIF1v3 play a major role in the crosstalk between BC and infiltrating immune cells mediating tumor progression, implying their high potential as target molecules for BC diagnostic, prognostication and treatment.

**Electronic supplementary material:**

The online version of this article (10.1186/s12935-018-0663-3) contains supplementary material, which is available to authorized users.

## Background

According to the latest statistics, females are more likely to develop breast cancer (BC) than any other cancer. It is the most commonly diagnosed cancer in more and less developed regions, and the second most common cause of cancer death in women worldwide [1]. BC initiation and progression are related to many factors including inflammatory factors which may be implicated in the development of therapy resistance [2].

During tumor development, the immune system can either recognize and destroy tumors or promote their growth. This process is called immunoediting [3]. Many studies have shown that the immune system is a major player in the cancer cell/tumor microenvironment crosstalk. Tumor-infiltrating immune cells are frequently observed and associated with cancer prognosis [4–7]. Several clinical studies have evaluated the prognostic significance of tumor-infiltrating lymphocytes (TILs) and tumor-associated macrophages (TAMs) in BC [8, 9]. Furthermore, inflammatory cytokines, such as interleukin 6 (IL-6) and tumor necrosis factor alpha (TNFα), have been shown to play important roles in the progression of BC [10].

Allograft inflammatory factor-1 (AIF1) was first identified in rat cardiac allografts undergoing chronic rejection [11]. In humans, the *AIF1* phylogenetically conserved gene is encoded within the major histocompatibility complex class III region on chromosome 6p21.3, which is known to harbor clusters of genes involved in inflammatory responses such as TNFα and nuclear factor-kappa B (NF-κB) [12]. Three splice isoforms have been identified including the AIF1 splice variant 3 (AIF1v3) considered to be the “wild-type” and the largest isoform encoding a 143–amino acid hydrophilic polypeptide of 17 kDa.

The function of AIF1 is not entirely known, but it has been found to be mainly expressed by immunocytes and closely associated with inflammatory diseases [13], obesity [14, 15], diabetes [16–18] and cancers [19, 20]. It is a well-known central mediator of inflammation by regulating the expression of inflammatory mediators such as cytokines, chemokines and inducible nitric oxide synthase [21, 22].

Indeed, numerous studies have demonstrated that AIF1 is involved in inflammatory responses, auto-immune diseases, reproductive immunity as well as immune activation and macrophage function [13]. AIF1 can increase IL-6, IL-10, and IL-12 production in the RAW 264.7 macrophage cell line stimulated with lipopolysaccharides [22]. In addition to immunomodulating functions, a recent report indicates that AIF1 may regulate several important cell adhesion molecules [23].

Previous studies have reported an increase in *AIF1* expression in malignancies and suggest that it may have a significant role in cancer progression [20, 24]. Furthermore, AIF1v3 may promote BC proliferation through activation of the NF-κB/cyclin D1 pathway [25]. Additional studies have shown that AIF1v3 may promote BC cell migration via the upregulation of TNFα-mediated activation of the p38-MAPK signaling pathway [26] and may increase the resistance of BC cells to cisplatin [27].

However, whether other AIF1 isoforms are also involved in BC development and progression has not yet been reported. The role and expression of AIF1 isoforms in the tumor microenvironment are also not known.

The present study aims to explore potential functions of two AIF1 isoforms (AIFv1 and AIF1v3) in breast tumors of varying severity and breast adipose tissue by evaluating their expression and relationship to metabolic and clinical parameters of BC patients. To better understand the association linking inflammation, AIF1 and BC progression, the relationship between components of the tumor inflammatory cell infiltrate and *AIF1* expression in breast adipose tissue was examined in histopathological breast tumor sections. The effect of omega-3 fatty acids (FA) on *AIF1* isoform expression was evaluated in lymphoblastoid cell lines (LCLs) cell lines to determine their potential functions.

Although AIF1 has been studied previously in other health conditions such as inflammatory diseases, to our knowledge, this is the first evaluating the AIF1v1 isoform in the context of BC.

## Methods

### Patients and study design

#### BRCAX population: AIF1

As part of a previous study, 115 women issued from BRCA1, BRCA2 and non-BRCA1/2 (BRCAX) families with high risk of BC were selected for transcriptome analysis. Selection of study population is described elsewhere [28]. Our BRCAX cohort comprised 16 pairs of BC affected and unaffected sisters issued from BRCAX families (age 60 ± 25 years). Using this cohort, we identified significantly and differentially expressed transcripts of the *AIF1* gene.

We characterized transcriptional profiles in French Canadian families with high risk of breast and ovarian cancer using RNA-sequencing methodology. RNA was isolated from immortalized LCLs of 115 women (affected and unaffected) issued from BRCA1, BRCA2 and BRCAX families. Statistical analyses were carried out using the R Package v3.3 as previously described [28]. Briefly, one-factor analysis of variance (ANOVA), Bonferroni correction and Scheffé’s test were performed to identify specific transcripts associated with the BRCAX subgroup. Overall, 190 transcripts were identified within BRCAX individuals, and two *AIF1* transcripts, in particular, were significantly and differentially expressed between the 16 pairs of affected and unaffected sisters. All individuals provided their written informed consent for the banking of their genetic material (Dr. J. Simard, director).

#### BC population and clinical evaluation

Our study population included pre-and postmenopausal women with BC that were prospectively recruited between January 2011 and May 2012, at the Deschênes-Fabia Center for Breast Diseases in Quebec City, Canada, a BC reference center. Briefly, women were eligible if they were not older than 70 years, were not pregnant, had no previous diagnosis of cancer other than non-melanoma skin cancer, never had any breast surgery, never took a selective estrogen receptor modulator, and had not received any treatment before BC surgery. Clinical data (data collection described previously [29]), and patient characteristics are shown in Table 1. Cohort 1, comprising thirteen women (age 48.3 ± 9.3 years, BMI 28.2 ± 6.8 kg/m^2^), was used to evaluate *AIF1* expression in breast tumors of varying severity. The expression of *AIF1* transcripts in breast adipose tissue was measured in cohort 2 comprising 74 women (age 51.9 ± 8.5 years, BMI 26.2 ± 5.6 kg/m^2^). All participants provided written informed consent. The study protocol was reviewed and approved by the Research Ethics Committee of the CHU de Quebec Research Center-Laval University, in accordance with their relevant guidelines and regulations.

**Table 1:** Description of the cohorts

Variables	Cohort 1 N = 13	Cohort 2 N = 74
Age^a^ (years)	48.3 ± 9.3	51.9 ± 8.5
BMI^a^ (kg/m^2^)	28.2 ± 6.8	26.2 ± 5.6
Menopausal status^b^		
Premenopause	8 (61.54)	17 (22.97)
Postmenopause	5 (38.46)	57 (77.03)
ER status^b^		
Positive	8 (61.54)	69 (93.24)
Negative	5 (38.46)	5 (6.76)
PR status^b^		
Positive	7 (53.85)	65 (87.84)
Negative	6 (46.15)	9 (12.16)
HER2 status^b^		
Positive	4 (30.77)	8 (10.81)
Negative	5 (38.46)	54 (72.97)
Not assessable	4 (30.77)	12 (16.22)
BC^b^		
DCIS	4 (30.77)	5 (6.76)
IDC		
Luminal	4 (30.77)	65 (87.84)
HER2+	2 (15.38)	1 (1.35)
Triple negative	3 (23.08)	1 (1.35)
Unclassified	–	2 (2.70)

*BMI* body mass index, *ER* estrogen receptor, *PR* progesterone receptor, *HER2* human epidermal growth factor receptor 2, *BC* breast cancer, *DCIS* ductal carcinoma in situ, *IDC* invasive ductal carcinoma

Data presented as ^a^mean ± SD, ^b^number of individuals (%)

#### Cell culture

Human cancer cell lines MCF7, ZR75, SKBR3, MDA-MB-231, BT20, OV90, OVCAR-3, LNCaP, HEK293, the non-cancerous human cell line MCF10A and the THP-1 monocytic human cell line were purchased from the American Type Culture Collection (ATCC). Adipocytes were isolated from mammary and abdominal (subcutaneous and omental) adipose tissue of women undergoing bariatric surgery. Fibroblasts were isolated from the mammary adipose tissue of BC patients. All cell lines except for THP-1, were subcultured according to ATCC recommendations.

THP-1 monocyte culturing and macrophage differentiation were carried out using Roswell Park Memorial Institute medium (RPMI 1640, Invitrogen) supplemented with 10% heat-inactivated FBS and 1% penicillin/streptomycin. Fibroblasts were maintained in Eagle’s Minimum Essential Medium supplemented with 15% FBS, 1% glutamine and 1% penicillin/streptomycin.

#### RNA isolation

RNA was extracted from monocytes, macrophages, adipocytes and fibroblasts using the miniRNeasy minikit (Qiagen). Total RNA from BRCA LCLs was extracted using TRI Reagent (Molecular Research Center Inc., Cincinnati, OH, USA) according to manufacturer’s instructions [29]. RNA was also obtained from human cancer cell lines (MCF7, ZR75, SKBR3, MDA-MB-231, BT20, OV90, OVCAR-3, LNCaP, HEK293) as well as BRCA LCLs. Samples of formalin-fixed and paraffin-embedded (FFPE) breast adipose tissue from BC patients were extracted with miRNeasy (FFPE) kit (Qiagen). BC samples of varying severity: DCIS and molecular subtypes of IDC, i.e. Luminal A/B (ER+ and/or PR+), HER2+ (ER−/PR−/HER2+) and triple-negative tumors (ER−/PR−/HER2−) were extracted using RNeasy FFPE kit (Qiagen). RNA was reverse-transcribed using the Superscript IV kit (Invitrogen). Non-FFPE samples were purified using the QIAquick PCR Purification Kit (Qiagen).

#### Monocyte culture and macrophage differentiation

Differentiation of human monocytic THP-1 cells into macrophages was performed according to Genin et al. [30]. Briefly, THP-1 monocytes were maintained in culture in RPMI medium and differentiated into macrophages following a 24 h incubation with 150 nM phorbol 12-myristate 13-acetate (PMA) followed by a 24 h incubation in RPMI medium. Macrophages were polarized to M1 macrophages by incubation for 24 h with 20 ng/ml of IFN-γ (Peprotech) and 10 pg/ml of LPS (Sigma). For M2 polarization, macrophages were incubated with 20 ng/ml of IL-4 (Peprotech) and 20 ng/ml of IL-13 (Peprotech) for 72 h. Cells were then collected using QIAzol and RNA was extracted as described.

#### EPA/DHA treatment

BRCAX LCLs were cultured in RPMI medium supplemented with 10% FBS and 1% penicillin/streptomycin in equally seeded 6-well plates, followed by incubation with either ethanol or various concentrations of eicosapentaenoic acid (EPA), docosahexaenoic acid (DHA), or a mixture of EPA: DHA (Sigma-Aldrich) for 24, 48 and 72 h ([EPA or DHA] = 0, 1, 5, 10, 20, 40, 50 µM and [EPA:DHA] = 5:5, 15:5 and 5:15 µM). Each omega-3 FA was dissolved by serially diluting in ethanol as recommended by the manufacturer and then added to media. RNA was then extracted, reverse-transcribed and purified. *AIF1* expression was quantified by qRT-PCR as described in the following section. All experiments were performed in triplicate, and similar results were obtained from each experiment.

#### Quantitative real-time PCR (qRT-PCR)

Oligoprimer pairs were designed by GeneTool 2.0 software (Biotools Inc, Edmonton, AB, CA) and their specificity was verified by blast in the GenBank database. The synthesis was performed by IDT (Integrated DNA Technology, Coralville, IA, USA) (see Additional file 1: Table S1).

cDNA corresponding to 20 ng of total RNA was used to perform fluorescent-based Realtime PCR quantification using the LightCycler 480 (Roche Diagnostics, Mannheim, DE). LightCycler 480 SYBRGreen I Master reagent (Roche Diagnostics, Indianapolis, IN, USA) was used with 2% DMSO, as described by the manufacturer. PCR was carried out using the following parameters: 45 cycles, denaturation at 98 °C for 10 s, annealing at 60 °C for 10 s, elongation at 72 °C for 10 s and then 72 °C for 5 s (reading). A melting curve analysis was performed to assess non-specific signals. Relative quantity was calculated using the fit point method and by applying the delta Ct method [31]. Normalization was done using reference genes that have shown stable expression levels from embryonic life through adulthood in various tissues [32]. ATP synthase subunit O (ATP5O), glucose-6-phosphate dehydrogenase (G6PD), hypoxanthine guanine phosphoribosyl transferase 1 (HPRT1) and glyceraldehyde-3-phosphate dehydrogenase (GAPDH) were used as reference genes for analysis in the breast adipose tissue. HPRT1 and GAPDH were used as reference genes for analysis in breast tumors, different cell types and BRCAX LCLs treated with DHA. qRT-PCR measurements were performed by the CHU de Québec Research Center (CHUL) Gene Expression Platform, Quebec, Canada and were compliant with MIQE guidelines [33, 34].

#### Assessment of tumor inflammatory infiltrate

Inflammatory cell reaction was assessed in haematoxylin and eosin (H&E) stained breast tumor sections from 15 BC patients, randomly selected according to their *AIF1v1* expression (n = 74) in breast adipose tissue (3 slides per patient, 45 slides total). Three methods were used to estimate the overall inflammation in each breast tumor section. First, the proportion of tumor cells and stroma within the delimited tumor area (Additional file 2: Figure S1A, B) were estimated semi-quantitatively. Tumor cell percentage (TCP) and tumor stroma percentage (TSP) were calculated as the percentage of the visible field comprised of tumor cells or stroma respectively, excluding other areas as described previously [7, 35].

Second, we scored peritumoral inflammatory cell infiltrate according to the Klintrup criteria (KM) [6]. Briefly, breast tumors were scored on a four-point scale based on their morphological appearance at the invasive margin (Additional file 2: Figure S1B). A KM score (KMS) of 0 was given when no increase of inflammatory cells was observed. A score of 1 denoted a mild and patchy increase of inflammatory cells, a score of 2 indicated a band-like infiltrate, and a score of 3 revealed a very prominent inflammatory reaction with frequent destruction of cancer cell islands. Inflammatory responses were subsequently classified as low grade (0/1) or high grade (2/3) for analysis. Lastly, to identify individual inflammatory cells and estimate their proportion within delimited tumor area, each section was divided into five distinct areas along the invasive margin as described previously [7]. Individual cells were counted at 20× magnification in 10 random boxes within each of the areas (0.018 mm^2^) resulting in a total of 50 boxes analyzed per slide. Boxes were randomly dispersed between the peritumoral and intratumoral regions. Cells outside of the tumor border, in necrotic areas and around normal lobules were excluded. Lymphocytes, plasma cells, eosinophils, macrophages and other cells (neutrophils and basophils) had characteristic morphological features that allowed them to be recognized and counted in H&E full-faced sections (representative box depicted in Additional file 2: Figure S1C). Additional parameters included tissue location, size of the delimited tumor area, tumor nest cells or stromal cells. Assessments of the tumor inflammatory infiltrate were performed by a single investigator (FAS) blinded to clinical and pathological data with independent co-scoring by a pathologist (KEI) to assess reproducibility. The inter-observer correlations were high: total cells (r > 0.99), lymphocytes (r > 0.99), plasma cells (r = 0.46), TCP (r = 0.78), TSP (r = 0.71) and KMS (r = 0.69).

### Statistical analyses

#### Association with clinical factors

Relationships between the expression of *AIF1* transcripts (in breast tumors and adipose tissue) with clinical and metabolic parameters including age (years), weight (kg), menopausal status, body mass index (BMI) (kg/m^2^), waist-hip ratio (WHR), adipose breast area (cm^2^) [36], inflammatory and hormonal gene expression measured in breast adipose tissue, were assessed using Spearman correlation. A p-value < 0.05 was considered statistically significant. Spearman correlations were adjusted for age without and with adiposity (BMI, weight or adipose breast area). All statistical analyses were performed with SAS software version 9.4 (SAS Institute Inc, Cary, NC, USA).

#### Bioinformatics analysis

AIF1v1 and AIF1v3 three-dimensional structures were constructed using the PDBsum program (EMBL-EBI) and vizualised with NGL Viewer application [37]. For multiple sequence alignment, the ClustalW2 server (EMBL-EBI) was used [38]. Predictive functions of each transcript were obtained with I-TASSER prediction server (ZhangLab) derived by threading the 3D models through protein function database BioLiP [39].

#### Other procedures

Protocols for MCF7 transfection and E1/E2 treatment, steroid extraction and 1D thin layer chromatography measurements, and crystal violet assay for determining viability of cultured cells are described in Additional file 3: Additional methods.

## Results

### Identification of *AIF1* transcripts in BRCAX families with high risk of BC

**Fig. 1:**
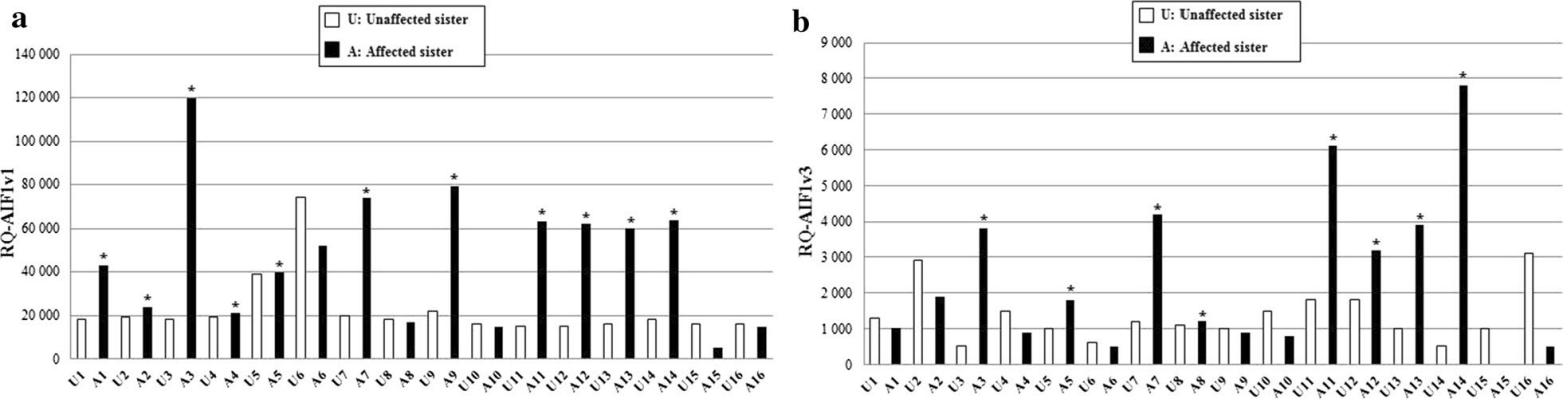
AIF1 gene expression analysis by RNA-sequencing in 16 pairs of BRCAX sisters (affected and unaffected) from French Canadian families with high-risk of BC. Overexpression of **a** AIF1v1 isoform and **b** AIF1v3 isoform in BRCAX LCLs of affected sisters compared to unaffected sisters. White bars correspond to unaffected sisters, black bars correspond to the BC affected sisters and asterisks (*) refer to significant increase in AIF1 expression between the affected and unaffected sister

Among a list of significant transcripts generated by RNA-sequencing and statistical analyses [28], specific transcripts were selected and validated by qRT-PCR to confirm their differential expression in BC affected and non-affected individuals in a BRCAX subgroup. The BRCAX cohort consists of 16 pairs of BC affected and unaffected sisters (one affected and one unaffected individual per family), the non-affected selected individual being the oldest sister of the family. Two splice variants of the *AIF1* gene (*AIF1v1* and *AIF1v3*) were identified as differentially expressed between the affected and unaffected sisters (Fig. 1). Indeed, *AIF1* expression in BRCAX immortalized LCLs was higher in the affected sisters compared to their unaffected sisters (*AIF1v1* overexpression in 11/16 pairs and *AIF1v3* overexpression in 8/16 pairs). These results were validated by qRT-PCR (Additional file 4: Figure S2A, B) and suggested that the two transcripts might be associated with BC; therefore, further investigation was warranted.

### Predictive structures, functions and interactions of AIF1 transcripts

Since little was known about AIF1, particularly AIF1v1, and its role BC development, the structure of the two variants was determined to examine potential functional differences.

**Fig. 2:**
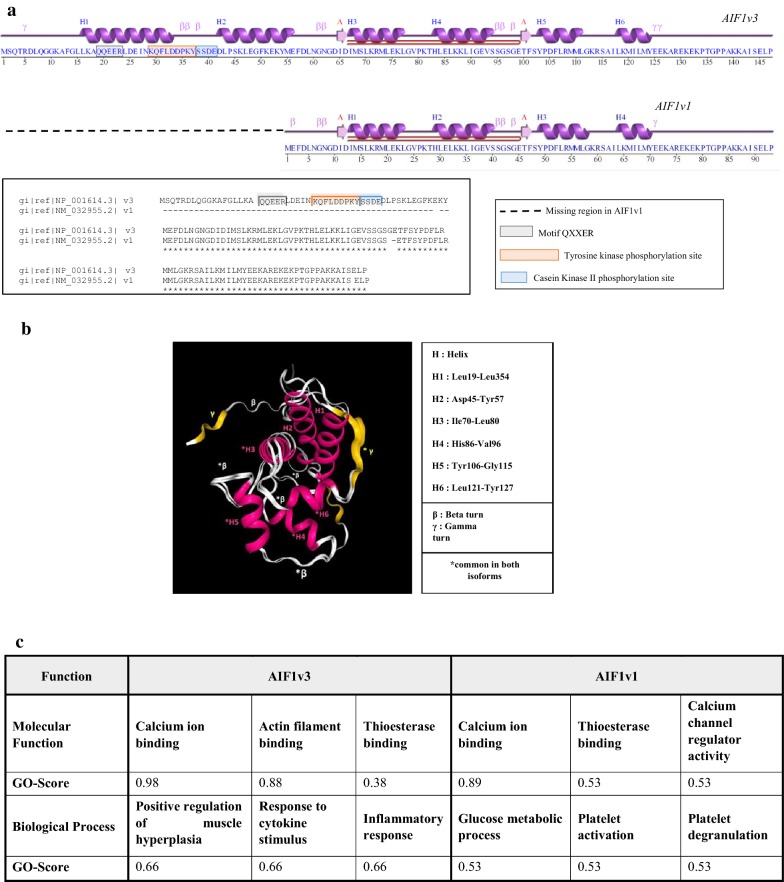
Predictive structure of AIF1 isoforms. **a** Representation of AIF1v3 and AIF1v1 secondary structures. Comparison of amino-acid sequences reveals a missing conserved region in isoform AIF1v1. Both isoforms share a leucine zipper motif that suggests possible dimerization of the corresponding proteins. Helices labelled H1, H2, H3, H4, H5 and H6 and strands by their sheets, labelled A. Motifs labelled β: beta turn, γ: gamma turn and beta hairpin (in red, A to A). **b** 3D model of AIF1v1 and AIF1v3 superpimposed. **c** Predictive protein functions. *GO* gene ontology. Consensus prediction derived based on the occurrence of the GO terms among the selected templates

As shown in Fig. 2a, structurally, AIF1v3 is the longest splice variant (147aa). AIF1v3 is the most extensively characterized of the two isoforms and is considered the “wild-type”. AIF1v1 is shorter (93aa) and shares with AIF1v3 a leucine zipper motif suggesting possible dimerization of the corresponding proteins. Both isoforms contain an EF-hand calcium-binding domain, the sequence pattern –KR–KK–GKR–, a motif typical of peptide hormone precursors, and are characterized by a wide range of biologically active sites. AIF1v3 contains a specific conserved region comprising the QXXER motif (19–23) that is important for G protein modulation and interactions involving synaptic transmission, a tyrosine kinase phosphorylation site (29–37) and a casein kinase II phosphorylation site (38–41) (a serine-threonine protein kinase with a wide range of substrates, many of which are involved in cell cycle regulation). This region is missing in AIF1v1 suggesting a specific functional role. 3D macromolecular models of both isoforms aligned with common sites highlighted are depicted in Fig. 2b.

Figure 2c reports predictions regarding molecular functions and biological processes of the two isoforms based on the occurrence of GO terms. While their molecular functions appear to be highly similar, the biological processes in which they are involved seem different. Indeed, AIF1v3 is more likely to be associated with the regulation of muscle hyperplasia and inflammatory responses, which is consistent with previous studies that identified this isoform as a crucial inflammatory mediator [14].

AIF1v1, on the other hand, seems to be involved in glucose metabolism and platelet activation suggesting a role in platelet glycolysis, but there is no data in the literature regarding this particular isoform. Glucose metabolism plays a critical role in the production, activation, and survival of platelets according to a recent study [40] and relationships between tumor and the hemostatic system is being recognized as an important BC regulator [41]. Considering these results, the potential roles and characterization of AIF1 were further explored.

### Characterization of AIF1 transcripts in breast tumors and human cancer cell lines

**Fig. 3:**
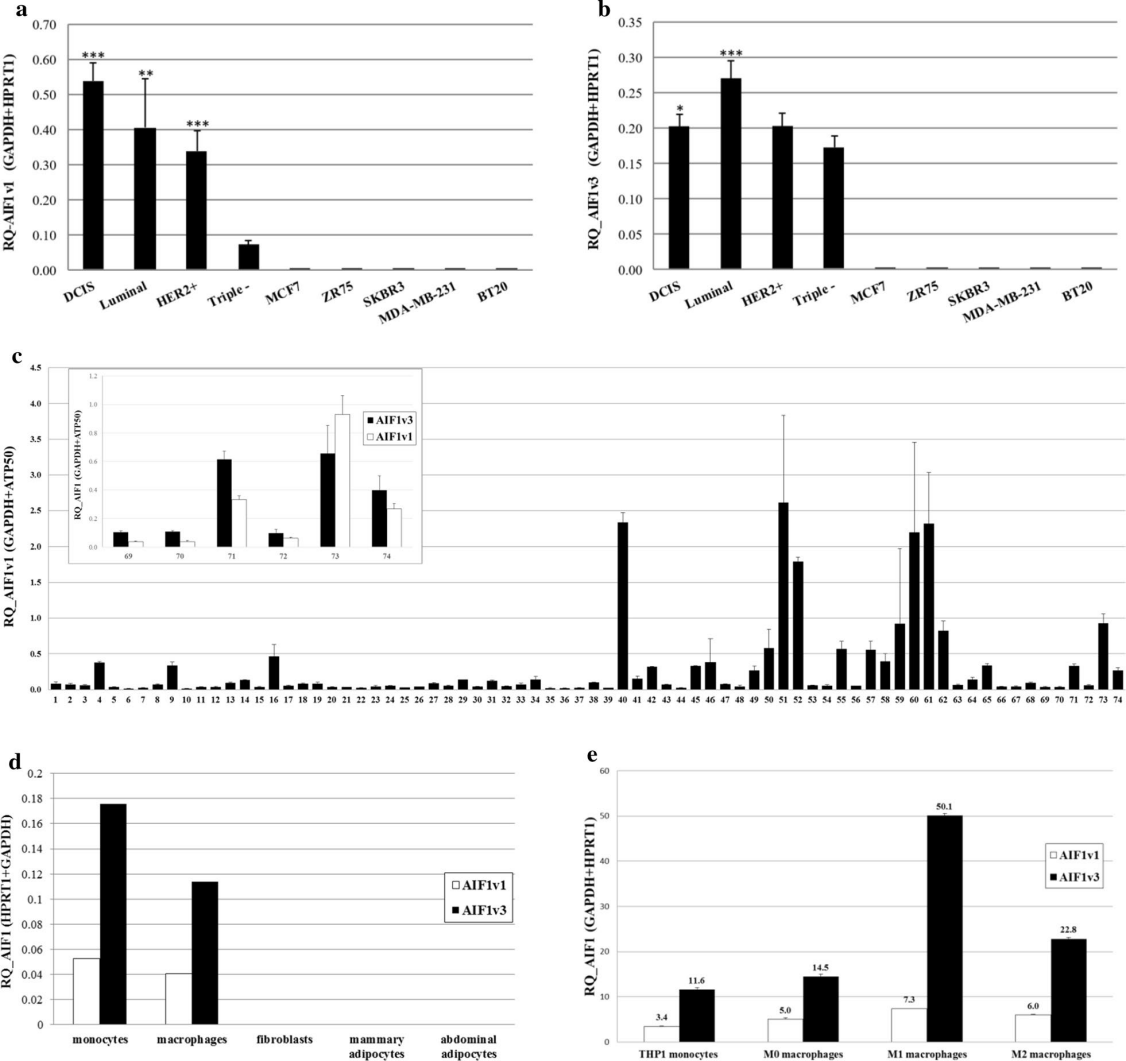
Characterization of AIF1 isoforms in breast tumors and tumor microenvironment. Expression of **a** AIF1v1 and **b** AIF1v3 in breast tumors of varying severity (DCIS, luminal A/B (ER+ and/or PR+), HER2+ (ER−/PR−/HER2+), triple negative (ER−/PR−/HER2−) and human BC cell lines (MCF7, ZR75, SKBR3, MDA-MB-231, BT20). **c** AIF1v1 in breast adipose tissue (top panel: comparison of AIF1v1 and AIF1v3 expression in six samples of breast adipose tissue). **d** AIF1 isoforms in various cell types of the breast tumor microenvironment (monocytes, macrophages, fibroblasts and adipocytes). **e** AIF1 isoforms in THP-1 monocytes and differentiated macrophages (M1, M2). Data shown as mean ± SD; Each subtype is compared to triple negative with ***p < 0.01, **p < 0.05, *0.05 < p < 0.1

Using cohort 1, presented in Table 1, gene expression analyses by qRT-PCR showed that both *AIF1v1* and *AIF1v3* isoforms are expressed in breast tumors of varying severity (Fig. 3a, b) and *AIF1v1* mRNA levels were higher than *AIF1v3*, considered the wild-type. Both isoforms seemed significantly more expressed in the less severe BC, i.e. ductal carcinoma in situ (DCIS) and luminal A/B (ER+ and/or PR+), and decreased with increasing BC severity, i.e. Her2+ (ER−/PR−/HER2+) and triple negative tumors (ER−/PR−/HER2−). This result suggests that AIF1 might be associated with BC initiation and progression. A similar experiment was conducted with 16 other breast tissues divided into benign structures, atypical ductal hyperplasia (ADH), DCIS and invasive ductal carcinoma (IDC). Results revealed little *AIF1v1* and *AIF1v3* expression in benign and ADH but were augmented in DCIS and IDC with expression rates significantly higher in DCIS (Additional file 5: Figure S3A, B). A decrease in cell viability was also observed in transfected MCF7 cells with *AIF1v1* as compared to controls (Additional file 6: Figure S4A, B). These findings corroborate our previous result showing increased expression of *AIF1* isoforms in breast tumors with the highest levels found in the less severe tumors (DCIS and luminal). However, *AIF1v3* seems to be more expressed in triple negative breast tumors (Fig. 3b) and IDC than *AIF1v1*. This finding suggests that these two isoforms behave differently and may perform different roles in BC development. No expression was observed in human BC cell lines MCF7, ZR75, SKBR3, MDA-MB-231, and BT20 (Fig. 3a, b), epithelial cancer cell lines OV90, OVCAR-3, LNCaP and HEK-293, and breast epithelial non-cancerous cell line MCF10A (Additional file 7: Figure S5A, B). This discovery implies that AIF1 is most probably not expressed in the epithelium and might rather be expressed via the tumor microenvironment (TME).

### Characterization of AIF1 transcripts in tumor microenvironment

To elucidate whether *AIF1* expression in breast tumors is due to the tumor microenvironment (TME), RNA was extracted from 74 samples of breast adipose tissue from BC patients and analyzed by qRT-PCR. Results showed that *AIF1v1* is highly expressed in breast adipose tissue, as well as *AIF1v3* (measured in 6 random samples), suggesting that AIF1 could be an important factor of the TME (Fig. 3c). Considering that major components of the breast adipose tissue include fibroblasts, adipocytes and immune cells, we further characterized cell types expressing the two isoforms. Figure 3d shows that *AIF1* is mostly expressed in monocytes and macrophages, particularly *AIF1v3*, but not in breast fibroblasts and adipocytes (mammary and abdominal). As two major polarization states have been described for macrophages, the classically activated type 1 (M1) and the alternatively activated type 2 (M2), we further investigated which type of macrophages express *AIF1*. The human monocytic cell line (THP-1) was differentiated into macrophages M1 and M2, and the expression of both isoforms was analyzed by qRT-PCR. *AIF1v3* was more strongly expressed in differentiated than undifferentiated macrophages, and this expression seemed higher in M1 than M2 macrophages (Fig. 3e). *AIF1v1* expression, however, was practically similar in differentiated and undifferentiated macrophages but slightly higher in M1. Results suggest that *AIF1v1* is probably mostly expressed in other cells of the breast TME.

### Assessment of tumor inflammatory infiltrate

As displayed in Table 2, the relationship between tumor inflammatory infiltrate in breast tumors and *AIF1v1* expression in corresponding breast adipose tissue was examined. Quantification of inflammatory cell reactions was assessed on full-section haematoxylin and eosin slides for 15 BC patients (3 slides per patient, 45 slides in total). Patients were clustered into three groups (tertiles) according to their *AIF1v1* expression levels (low, med, high). Tumor cell percentage (TCP) and tumor stroma percentage (TSP) were similar in each group and no significant differences were observed. Using the Klintrup–Mäkinen (KM) criteria, the peritumoral inflammatory cell response was graded as “low KM score” in 8 BC patients (53.33%) and “high KM score” in 7 BC patients (46.67%). In general, all patients in the high *AIF1v1* expression group had a high KM score while 80% of patients in med and low groups had a low KM score.

**Table 2:** Distribution of the infiltrating immune cells, KM scores, TSP, TCP and NP between the different *AIF1v1* expression groups in breast tumors

Variables	*AIF1v1* expression			p-value
	Low (n = 5)	Med (n = 5)	High (n = 5)	
Total cells count^a^	255.7 ± 95.5	327.7 ± 215.4	1108.6 ± 847.5	0.03*
Lymphocytes count^a^	251.1 ± 98.5	321.9 ± 218.4	1095.2 ± 838.6	0.03*
Plasmocytes count^a^	2.8 ± 1.5	4.0 ± 3.8	8.3 ± 2.7	0.02*
TCP^b^	67.8 ± 11.8	60.0 ± 8.0	64.1 ± 8.4	0.46*
TSP^b^	32.2 ± 11.8	40.0 ± 8.0	35.9 ± 8.4	0.46*
KMS high^c^	1 (20%)	1 (20%)	5 (100%)	0.02**

*TCP* tumor cell percentage, *TSP* tumor stroma percentage, *KMS* Klintrup and Mäkinen score

* Analysis of variance using one-way ANOVA. ** Fisher’s Exact test. Italic text indicates a statistically significant difference with a p-value < 0.05

Data presented as mean ^a^number of cells, ^b^percentage per group of patients ± SD and ^c^number of individuals (%)

**Fig. 4:**
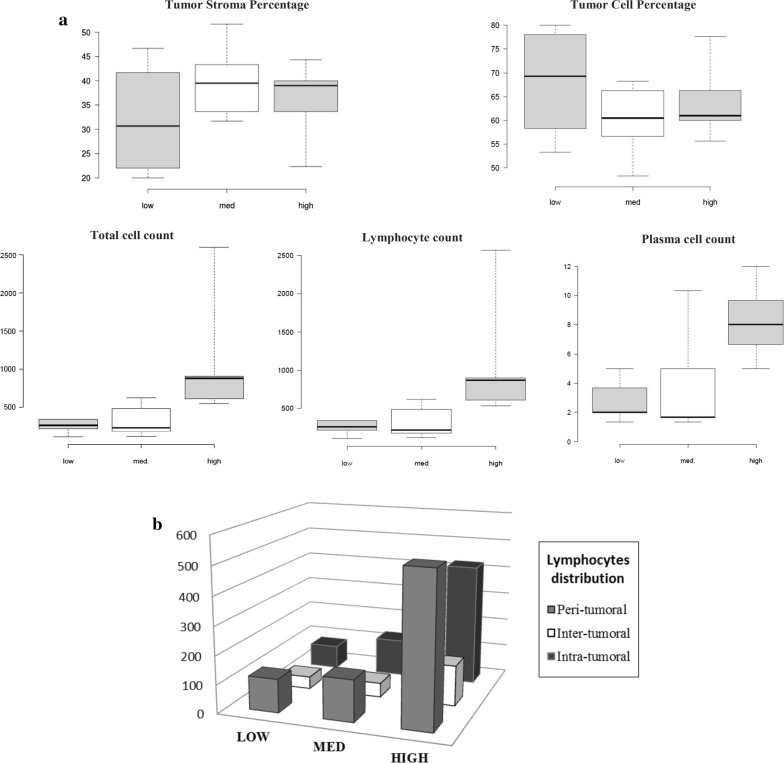
Assessment of tumor inflammatory infiltrate in breast tumors. **a** Boxplot representation of the relationships between adipose AIF1v1 expression and tumor stroma percentage (TSP), tumor cell percentage (TCP), total cells (p = 0.03, ANOVA), lymphocytes (p = 0.03, ANOVA) and plasma cells (p = 0.02, ANOVA). **b** Distribution of total lymphocytes in peri, inter and intratumoral areas. Individuals clustered in tertiles: first group (low) consisted of BC patients with the lowest AIF1v1 rates in breast adipose tissue, the second was intermediate (med) and the third, the highest (high)

Patients who overexpressed *AIF1v1* also had a higher number of total inflammatory cells while med and low groups had significantly lower cell numbers (Fig. 4a). Lymphocytes and plasma cells were primarily identified with relatively few eosinophils, macrophages or other cell types (these cell types were excluded from further analysis). Lymphocytes were the most abundant cells in all groups and were equally distributed between peritumoral and intratumoral regions (Fig. 4b). Furthermore, significant correlations were found between breast adipose *AIF1v1* expression with total number of lymphocytes (rs = 0.63; p = 0.01), number of intratumoral lymphocytes (rs = 0.80, p = 0.0006), number of peritumoral lymphocytes (rs = 0.58, p = 0.03) and total number of plasma cells (rs = 0.67, p = 0.005), respectively.

Overall, BC patients with high *AIF1v1* breast adipose expression had more cell infiltrate at the invasive margin (KM score) and a significantly higher number of infiltrating immune cells consisting predominantly of lymphocytes, both in the peri and intra-tumoral regions.

### Functional role of AIF1

To further investigate potential functions of AIF1 and taking into consideration its high expression in breast adipose tissue, we measured the conversion rate of ^14^C-estradiol (E2) to estrone (E1) (and inversely) in transfected MCF7 cell lines. The results revealed that the estrone/17beta-estradiol conversion rate, regardless of E2/E1 concentration and incubation time, was similar in MCF7 cells (control) and transfected MCF7 cells with pcDNAv1 and pcDNAv3 (Additional file 8: Figure S6) demonstrating that AIF1v1 and AIF1v3 do not intervene in the E2/E1 conversion rate.

**Fig. 5:**
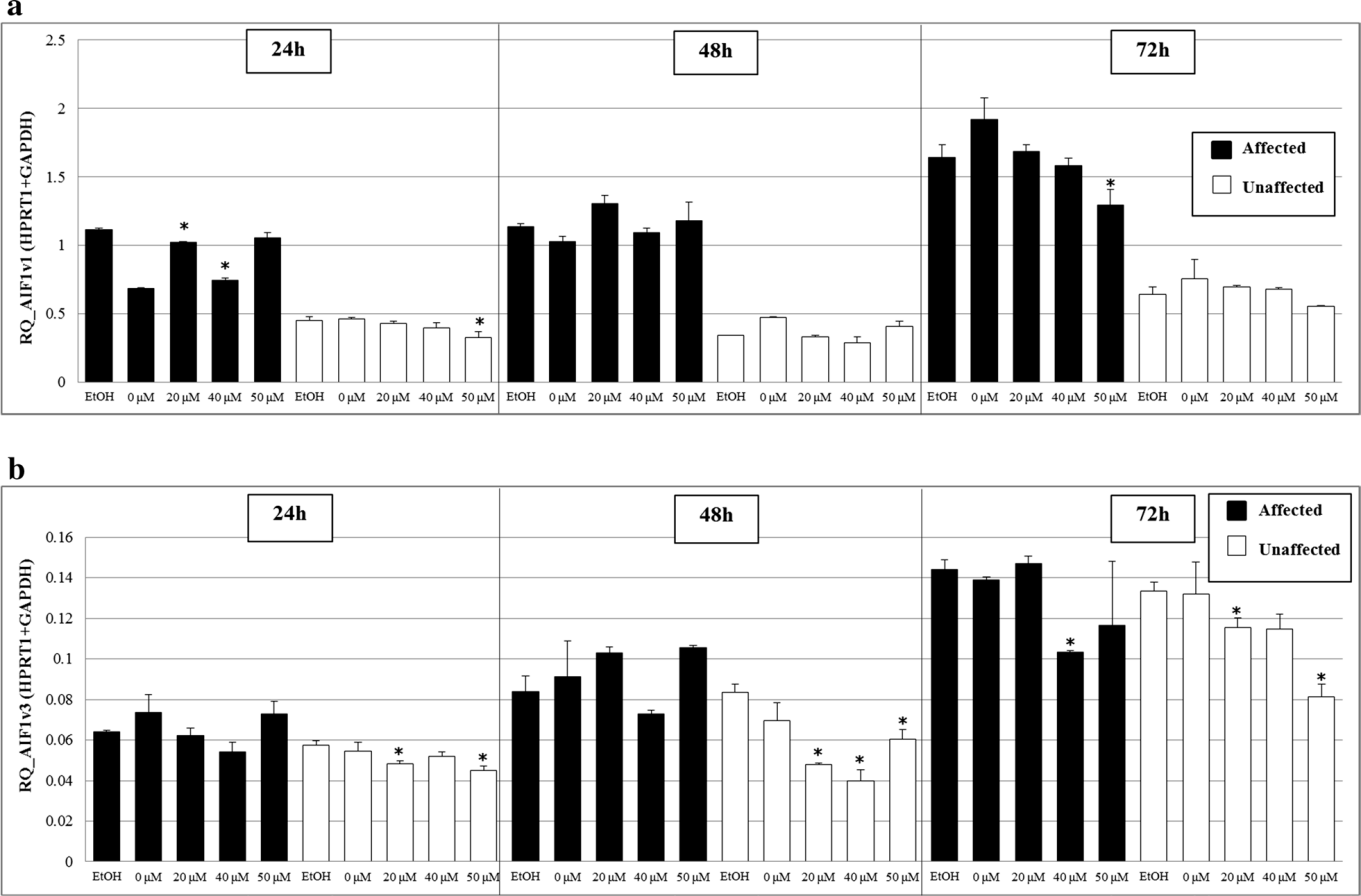
Effect of long-chain omega-3 fatty acid docosahexaenoic acid (DHA) on the expression of **a** AIF1v1 and **b** AIF1v3. BRCAX LCLs of affected and unaffected sisters were treated with 0, 20, 40 and 50 µM of DHA for 24, 48 and 72 h. Data shown as mean ± SD. Significant values are highlighted with asterisks (*), each sample was compared to control (ethanol)

Given its implication in inflammation, the potential biological process in which AIF1 could intervene in the metabolic pathway of polyunsaturated fatty acids (PUFAs) was investigated. Long-chain omega-3 FA eicosapentaenoic acid (EPA) and DHA are important in generating bioactive lipid mediators that are necessary for inflammation resolution [42]. Analyses of BRCAX immortalized LCLs supplemented with various concentrations of EPA, or a mixture of EPA: DHA for 24 h showed a significant decrease in *AIF1v1* and *AIF1v3* expression when cells were incubated with 40 µM of DHA (p < 0.05) for both the affected and unaffected BRCAX sisters (Additional file 9: Figure S7A, B). An independent assay performed with incubation of BRCAX LCLs of the same sister pair with a higher DHA concentration and over a longer period revealed that *AIF1v1* and *AIF1v3* expression levels were consistently lower after DHA supplementation (Fig. 5). The decrease persisted over time and was dose-dependent.

### AIF1 expression and clinical parameters of BC patients

Using our cohorts presented in Table 1, *AIF1* expression in breast tumors (n = 13) and breast adipose tissue (n = 74) was then investigated in relation to clinical parameters of BC patients (Table 3).

**Table 3:** Spearman’s correlation between *AIF1v1/v3* expression in breast tumors and breast adipose tissue and variables

Variable	N	Breast tumors		N	Breast adipose tissue	
		*AIF1v1* expression	*AIF1v3* expression		*AIF1v1* expression	*AIF1v1* expression adjusted*
		Rs (p)	Rs (p)		Rs (p)	Rs (p)
Age (years)	13	***0.65 (0.02)***	*0.52 (0.06)*	74	0.16 (0.16)	**–**
Weight (kg)	13	0.05 (0.85)	0.17 (0.58)	74	***0.25 (0.03)***	*0.22 (0.06)*
BMI (kg/m^2^)	13	0.22 (0.47)	0.30 (0.32)	74	*0.19 (0.09)*	0.16 (0.16)
Waist-hip ratio	13	0.33 (0.27)	0.37 (0.21)	74	*0.23 (0.05)*	0.17 (0.14)
Menopausal status	13	*0.55 (0.05)*	0.08 (0.78)	74	*0.19 (0.09)*	0.06 (0.57)
Adipose breast area (cm^2^)	13	0.32 (0.28)	0.27 (0.36)	74	***0.31 (0.008)***	***0.27 (0.02)***
CYP19A1 expression	12	***0.63 (0.03)***	0.39 (0.21)	74	***0.83 (< 0.0001)***	***0.82 (< 0.0001)***
ERα expression	12	0.37 (0.24)	0.08 (0.79)	74	***0.49 (< 0.0001)***	***0.47 (< 0.0001)***
Leptin expression	7	0.39 (0.38)	0.21 (0.64)	74	***0.63 (< 0.0001)***	***0.62 (< 0.0001)***
COX2 expression	7	0.61 (0.15)	***0.79 (0.04)***	73	***0.40 (0.0005)***	***0.40 (0.0006)***
IL-6 expression	7	***0.89 (0.006)***	0.64 (0.12)	74	***0.60 (< 0.0001)***	***0.59 (< 0.0001)***
TNFα expression	6	0.08 (0.87)	− 0.26 (0.62)	64	***0.45 (0.0002)***	***0.47 (< 0.0001)***

*Rs* Spearman correlation coefficient, *p* p-value, *BMI* body mass index, *CYP19A1* Cytochrome P450 Family 19 Subfamily A Member 1, *Erα* estrogen receptor alpha, *COX2* cyclooxygenase 2, *IL-6* interleukin 6, *TNFα* tumor necrosis factor alpha

Bold italics text indicates a statistically significant difference with a p-value < 0.05, text in italics shows a p-value between 0.05 and 0.1. Adipose breast area is estimated from mammograms [36]

* Adjusted for age when applicable

*AIF1v1* expression in breast tumors significantly and positively correlated with age (p = 0.02), menopausal status (p = 0.05) and breast adipose expression of Cytochrome P450 Family 19 Subfamily A Member 1 (CYP19A1) (p = 0.03) and IL-6 (p = 0.006) whereas *AIF1v3* was only positively correlated with breast adipose expression of cyclooxygenase-2 (COX2) (p = 0.04). Expression of each isoform in breast tumors also correlated with each other (r = 0.63; p = 0.02).

In breast adipose tissue, *AIF1v1* expression significantly and positively correlated with weight (p = 0.03), waist-hip ratio (WHR) (p = 0.05), adipose breast area (p = 0.008) and breast adipose expression of CYP19A1 (p < 0.0001), estrogen receptor alpha (ERα) (p < 0.0001), leptin (p < 0.0001), COX2 (p < 0.0005), IL-6 (p < 0.0001) and TNFα (p = 0.0002). When adjusted for age, these correlations were still highly significant for all variables except weight (p = 0.06) and WHR (p = 0.14). These correlations were also highly significant when adjusting for age and adiposity (body mass index (BMI), weight or adipose breast area).

Similarly to Fig. 3a, *AIF1v1* was highly expressed in the less severe tumors and decreased with increasing disease severity when measured in the breast adipose tissue (Additional file 10: Figure S8).

These results highlight the importance of AIF1v1 isoform in BC and provide evidence of its interaction with a series of proinflammatory cytokines such as IL-6 and TNFα, adipokines such as leptin and other important variables that play key roles in BC progression.

## Discussion

AIF1 is a cytoplasmic, EF-hand calcium-binding, inflammation-responsive scaffold protein that is implicated in various disease processes. Originally identified in chronically rejecting cardiac allografts, it was initially demonstrated that AIF1 was a modulator of the immune response [43]. Previous reports have described a range of AIF1-related splice variants, and three splice isoforms have been identified: AIF1v1, AIF1v2 and AIF1v3 (GenBank accession nos. NM_032955, NM_004847 and NM_001623, respectively) but to date, there are no studies published on AIF1v1. In the present study, we investigated, for the first time, the functional and structural differences of AIF1v1 and AIF1v3, and their expression in breast tumors and breast tumor microenvironment. We identified AIF1 isoforms in a cohort of BRCAX individuals issued from families with high BC risk. In these families, *AIF1v1* and *AIF1v3* were significantly and differentially expressed between BC affected and unaffected sisters within the same families, which led us to investigate their implication in BC development.

Cells transfected with AIF1v1 showed reduced cell viability, in agreement with a previous study with AIF1 in pancreatic cells [17]. In addition, both *AIF1* isoforms, but mainly *AIF1v1*, were highly expressed in the less severe BC tumors (DCIS and luminal subtype) suggesting their involvement in tumor initiation and progression. Two previous studies demonstrated that AIF1v3 in transfected human BC cell lines could promote BC cell proliferation via the NF-κB pathway and enhance cell migration by activation of p38-MAPK pathway suggesting a possible role in BC progression [25, 26]. However, roles of both isoforms and interactions with other proteins in the tumor microenvironment have not been elucidated.

Our data revealed that while AIF1 isoforms are not present in epithelial cells, they are highly expressed in breast adipose tissue. Furthermore, we observed large differences in expression rates and isoform sources. While activated macrophages are the major source of *AIF1v3* in breast adipose tissue, which is consistent with previous findings in human white adipose tissue [15], *AIF1v1* appears to be significantly less expressed by macrophages, suggesting that its high expression is due to another immune cell type. Since BRCAX LCLs displayed high *AIF1v1* mRNA expression levels, we hypothesized that lymphocytes might be a major source. The latter result is supported by a previous study where several T-cell lines were screened by real-time PCR to compare *AIF1* isoform expression levels with those of peripheral blood mononuclear cells and showed a higher expression of the *AIF1v1* isoform in all immortalized cell lines screened [44].

Furthermore, bioinformatics analyses showed a significant structural difference between both isoforms. AIF1v1 appears to lack an entire region including specific conserved motifs and binding sites that are present in AIF1v3. This observation is of substantial relevance and could explain why the two isoforms may be differentially expressed by various cell types regulated by cytokines and growth factors of the environment and thus, behave differently. Previous studies demonstrated that *AIF1v3* is strongly expressed in macrophages and activated T-cells [19, 44, 45], but no data exists on *AIF1v1*. TILs and TAMs have essential roles in mediating tumor progression in all BC subtypes. TAMs have been shown to possess features of the pro-inflammatory M1 phenotype in the early stages of tumorigenesis, but switch to an anti-inflammatory M2-like phenotype, with the acquisition of proangiogenic capability [46]. Furthermore, IL-6 and other cytokines secreted by M1-polarized macrophages have been shown to be involved in a wide range of tumorigenic processes [47].

This is concordant with our results showing that *AIF1v3* is more highly expressed in M1 than M2 macrophages, its expression being the highest in DCIS and luminal, and decreasing as BC prognostic severity increases. A recent study showed that RAW264.7 cells overexpressing *AIF1v3* increased markers associated with M2 polarization and decreased those associated with M1 polarization [48]. However, these results were carried out in a specific transfected subset of colony-stimulating factor (CSF1)-induced macrophages in the context of hepatocellular carcinoma and not BC. In addition, M2 polarization is a complex process that involves multiple factors other than CSF1, such as monocyte chemoattractant protein-1 [49]. Nevertheless, in our case, *AIF1v3* was expressed in both M1 and M2 macrophages with significantly higher expression in the M1 phenotype.

In one retrospective study of 53 mastectomy samples, increased B-cell and T-cell immune infiltrate was identified in benign ductal hyperplasia and was increased in DCIS and highest in invasive BC [50]. This suggests that a particular class of lymphocytes is responsible for its expression in these particular BC subtypes. Two TILs phenotypes have been described: type 1, which is assumed to have anti-tumor properties and type 2, which may promote an anti-inflammatory immune response that could enhance tumor growth [51, 52]. The distribution of these different types of lymphocytes in each BC subtype needs to be further investigated. Furthermore, whether *AIF1v1* is only expressed by a particular class of lymphocytes requires further study.

Previous studies have established that cancer development and progression depend on complex interactions between the tumor and the local inflammatory response [53], and a number of immune cell types implicated in this response have been described [4, 5, 7, 54]. Our assessment of the tumor inflammatory cells infiltrate in breast tumors showed that adipose AIF1v1 was associated with the number of lymphocytes infiltrating breast tumors in both the peri and intra-tumoral regions and total number of plasma cells, which allow us to confirm our previous hypothesis. It has been reported that the prognostic significance of tumor-infiltrating T cells in breast carcinoma depends on their relative density and tissue location (peri or intra-tumoral) [55]. Given the functional heterogeneity of TILs, the link between the tissue location of TILs infiltrate and *AIF1v1* expression would need to be further investigated.

It is well known that estrogens, which are expressed in many immune cells, modulate inflammatory cytokine gene expression [56–59]. A previous study reported that E2 increased *AIF1v3* expression in a RAW264.7 murine macrophage cell line [60]. However, our analysis showed that AIF1v3, as well as AIF1v1, do not interfere with the biological activity of estrogens in MCF7 cells thus the effect of estrogens on AIF1 is likely due, in part, to another mechanism.

As for omega-3 FA, it has been reported that they decrease cell proliferation and induce apoptotic cell death in human BC cells through the NF-κB cell pathway [61], and it is established that AIF1 promotes BC proliferation via activation of the NF-κB/cyclin D1 pathway [25]. This may explain our results showing the ability of DHA to modulate *AIF1v1* and *AIF1v3* expression in BRCAX LCLs in a dose-dependent manner. This is of interest because it shows for the first time that omega-3 FA, namely DHA, may potentially work as adjuvants and safe complementary therapies to standard cancer treatment [62–64], and prevent tumor growth and progression by reducing *AIF1v1* and *AIF1v3* expression in BC patients, particularly those exhibiting less aggressive tumors.

Concerning clinical and metabolic phenotypes in BC patients, AIF1v1 was significantly correlated with age and menopausal status in breast tumors. Since this is the first time these relations have been observed, the influence of menopause on AIF1v1 deserves further investigation. AIF1 was also positively correlated with weight, WHR and adipose breast area in mammograms. These significant correlations with adipose *AIF1* expression are in agreement with previous findings suggesting AIF1 is an adipokine associated with clinical parameters related to obesity [15]. The fact that *AIF1v1* expression was strongly associated with CYP19A1, leptin and ERα shows that it is involved in this pathway and plays a significant role in adipose-inflammation induced BC. Indeed, CYP19A1 provides instructions for making an enzyme called aromatase. Aromatase expression and activity in the breast adipose tissue is upregulated by leptin and inflammatory mediators and is associated with increased tissue levels of COX2 and prostaglandin E2 (PGE2) [65]. The upregulation and associated effects can drive aberrant estrogen production within the mammary tissue, thereby promoting BC tumorigenesis. Finally, its association with inflammatory factors such as COX2, IL-6 and TNFα provides further evidence that AIF1v1 is a key regulator of inflammation in the breast tumor microenvironment and interacts with a wide variety of cytokines and adipokines.

Taken together, these results imply that AIF1v1 can potentially regulate the recruitment and activation of inflammatory cells, particularly lymphocytes, and redirect the immune response to promote the construction of a microenvironment that is more suitable for breast cancer cell progression. The underlying mechanism is yet to be elucidated. However, we can hypothesize it implies the production of TNFα by AIF1v1-activated lymphocytes, which will lead to the activation of NF-κB, thereby promoting the production of IL-6 and other cytokines, and growth-factor signals.

## Conclusion

Our results shed some light on the importance of the AIF1v1 isoform and its role in breast tumor progression. Given that AIF1 in highly expressed in the less severe breast tumors, it may prove useful as a favorable prognostic factor for BC. We also provide pertinent information on how both AIF1 isoforms relate to the breast tumor microenvironment. Importantly, we have shown that DHA can potentially decrease AIF1 expression, which may reduce inflammation-induced BC. The highly significant results obtained in our patient cohorts need to be further investigated to evaluate whether AIF1 may be useful as a therapeutic target for BC.

## Additional files

**Additional file 1: Table S1.** Primer sequence and gene description.

**Additional file 2: Figure S1.** Estimation of inflammation reaction methods. (A) Delimitation of tumor area and estimation of tumor cell percentage (TCP) and tumor stroma percentage (TSP); (B) Scoring of general inflammatory infiltrate at the invasive margin (Klintrup criteria); (C) Representation of inflammatory cell counting at 20× magnification in one random box in the breast tumor (0.018 mm^2^).

**Additional file 3.** Additional methods.

**Additional file 4: Figure S2.** Validation of AIF1 expression in BRCAX immortalized lymphoblastoid cells (LCLs) by qRT-PCR in (A) affected sister and (B) non-affected sister. A = affected; NA = non-affected.

**Additional file 5: Figure S3.** Expression of AIF1 in mammary tissue in isoforms (A) AIF1v1 and (B) AIF1v3. ADH = Atypical ductal hyperplasia; DCIS = Ductal carcinoma in situ; IDC = Invasive ductal carcinoma.

**Additional file 6: Figure S4.** (A) Analysis of cell viability by crystal violet staining was performed on equal numbers of MCF7 breast cancer cells plated in a 12-well cell culture dish. The cells were transfected with (1) MCF7 alone (2) transfection agent (jetPRIME) 3) empty vector (pcDNA3.1 (+)) and (4) pcDNA3.1 (+)-AIF1v1 and let grown for 4 days. The purple color reflects the number of colonies formed after 4 days. A decrease in the number of colonies indicates decreased proliferation or increased cell death in presence of AIF1. (B) Relative expression levels of AIF1v1 mRNA by real-time PCR. The MCF7 cells seeded in 12-well plates were transfected with (1) MCF7 alone (2) transfection agent (jetPRIME) (3) empty vector (pcDNA3.1(+)) and (4) pcDNA3.1(+)-AIF1v1. HPRT1 was used as an internal control.

**Additional file 7: Figure S5.** Expression of AIF1v1 (A) and AIF1v3 (B) in cancer cell lines.

**Additional file 8: Figure S6.** Conversion of E1/E2 at different incubation time periods. E1 = Estrone; E2 = ^14^C-estradiol.

**Additional file 9: Figure S7.** AIF1v1 (A) and AIF1v3 (B) expression at varying concentrations of EPA/DHA EPA = Eicosapentaenoic acid; DHA = Docosahexaenoic acid.

**Additional file 10: Figure S8.** Distribution of breast adipose AIF1v1 expression in BC patients diagnosed with various breast tumors: ductal carcinoma in situ (DCIS), luminal A/B (ER+ and/or PR+), HER2+ (ER−/PR−/HER2+) and triple negative (ER−/PR−/HER2).

## Abbreviations

ADH: atypical ductal hyperplasia
AIF1: allograft-inflammatory factor-1
ATCC: American Type Culture Collection
ATP5O: ATP synthase subunit O
BC: breast cancer
BMI: body mass index
COX2: cyclooxygenase-2
CSF1: colony-stimulating factor 1
CYP19A1: Cytochrome P450 Family 19 Subfamily A Member 1
DCIS: ductal carcinoma in situ
DHA: docosahexaenoic acid
E1: estrone
E2: ^14^C-estradiol
EPA: eicosapentaenoic acid
ER: estrogen receptor
ERα: estrogen receptor alpha
FFPE: formalin-fixed and paraffin-embedded
GAPDH: glyceraldehyde-3-phosphate dehydrogenase
G6PD: glucose-6-phosphate dehydrogenase
HER2: human epidermal growth factor receptor 2
H&E: haematoxylin and eosin
HPRT1: hypoxanthine guanine phosphoribosyl transferase 1
IDC: invasive ductal carcinoma
IL-6: interleukin-6
KM: Klintrup–Mäkinen criteria
KMS: KM score
LCLs: lymphoblastoid cells
NF-κB: nuclear factor-kappa B
PGE2: prostaglandin E2
PMA: phorbol 12-myristate 13-acetate
PR: progesterone receptor
PUFAs: polyunsaturated fatty acids
TAMs: tumor-associated macrophages
TCP: tumor cell percentage
TILs: tumor-infiltrating lymphocytes
TME: tumor microenvironment
TNFα: tumor necrosis factor alpha
TSP: tumor stroma percentage
WHR: waist–hip ratio
