# Supplementary material for: An isoform of AIF1 involved in breast cancer
Source: Cancer Cell Int. 2018 Oct 22;18:167. doi: 10.1186/s12935-018-0663-3 (PMC6198497; doi:10.1186/s12935-018-0663-3)
Supplement: Supplementary file 1 — Additional file 1: Table S1. Primer sequence and gene description. [file 12935_2018_663_MOESM1_ESM.docx]

Table S1. Primer sequences and gene description

| Gene Symbol | Description | GenBank | size  (pb) | Primer sequence 5'→3'  S/AS |
| --- | --- | --- | --- | --- |
| AIF1v1 | Homo sapiens allograft inflammatory factor 1 (AIF1), transcript variant 1 | NM_032955 | 143 | CGTTGTCTCCTCCACCTAGCAGTT/TAT CGCCATTTCCATTAAGGTCAA |
| AIF1v3 | Homo sapiens allograft inflammatory factor 1 (AIF1), transcript variant 3 | NM_001623 | 185 | AACCAGGGATTTACAGGGAGGAA/TAT CGCCATTTCCATTAAGGTCAA |
| HPRT1 | Homo sapiens hypoxanthine phosphoribosyltransferase 1 | NM_000194 | 157 | AGTTCTGTGGCCATCTGCTTAGTAG/AA ACAACAATCCGCCCAAAGG |
| GAPDH | Homo sapiens glyceraldehyde-3-phosphate dehydrogenase | NM_002046 | 194 | GGCTCTCCAGAACATCATCCCT/ACGCC TGCTTCACCACCTTCTT |
| ADNg | Homo sapiens 3-beta-hydroxysteroid dehydrogenase/delta-5-delta-4-isomerase (3beta-HSD) gene (intron) | M38180 | 260 | GAAGGGCAGAGGTGGAACTAGAA/AAC AAAGACCAAAGACCAGTGAGA |
| CYP19A1 | Homo sapiens cytochrome P450, family 19, subfamily A, polypeptide 1, 2 transcripts | NM_000103 | 57 | CCAGTGAAAAAGGGGACAAACATTA/C TCGAGTCTGTGCATCCTTCCAATA |
| ERα | Homo sapiens estrogen receptor 1, 6 transcripts | NM_000125 | 87 | TGCAAAATCTAACCCCTAAGGAAGTG/ ATGGGCTATGGCTTGGTTAAAC |
| Leptin | Homo sapiens leptin (LEP) | NM_000230 | 64 | TTTTGTCAAGTGTCATATGTAGGTGTC/ CTCCCTTCTGCCCAAACATTC |
| COX2 | Homo sapiens prostaglandin-endoperoxide synthase 2 (prostaglandin G/H synthase and cyclooxygenase) (PTGS2) | NM_000963 | 75 | TGCAATAACGTGAAGGGCTGTC/GATG GTGACTGTTTTAATGAGCTCTG |
| IL-6 | Homo sapiens interleukin 6 (interferon, beta 2) | NM_000600 | 70 | CTGGTGTTGCCTGCTGCCTTC/GTGGGG CGGCTACATCTTTGG |
| TNFα | Homo sapiens tumor necrosis factor | NM_000594 | 85 | CATCAAGAGCCCCTGCCAGAG/GAAGA CCCCTCCCAGATAGATG |
| ATP5O | Homo sapiens ATP synthase, H+ transporting, mitochondrial F1 complex, O subunit | NM_001697 | 103 | ATTGAAGGTCGCTATGCCACAG/CCTTC AGGATTTGTGCTACTCTCA |
| G6PD | Homo sapiens glucose-6-phosphate  dehydrogenase (G6PD), nuclear gene encoding mitochondrial protein | NM_000402 | 77 | GCCAACCGCCTCTTCTACCTG/ATGCAG GACTCGTGAATGTTCTTG |
