# Supplementary figures and images for: An isoform of AIF1 involved in breast cancer
Source: Cancer Cell Int. 2018 Oct 22;18:167. doi: 10.1186/s12935-018-0663-3 (PMC6198497; doi:10.1186/s12935-018-0663-3)

Figure S1

A)

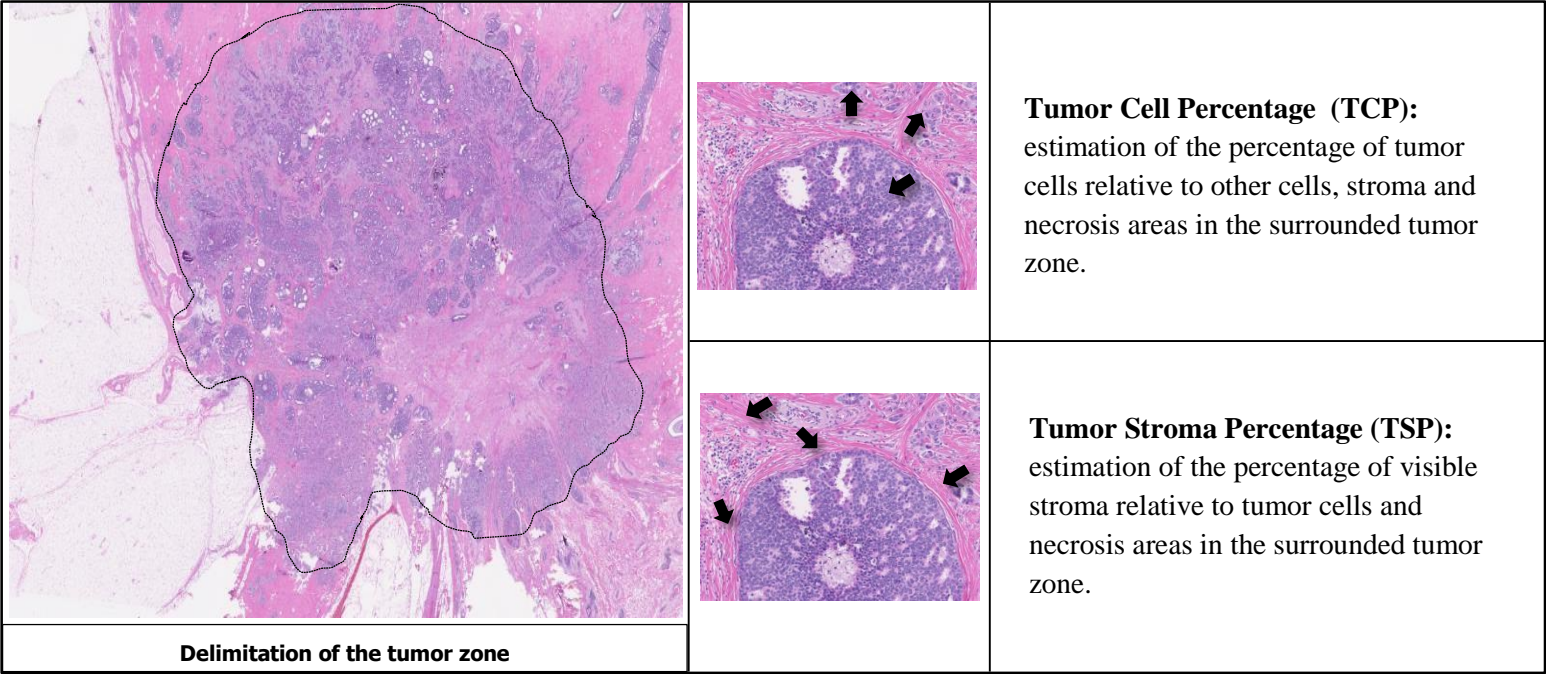

B)

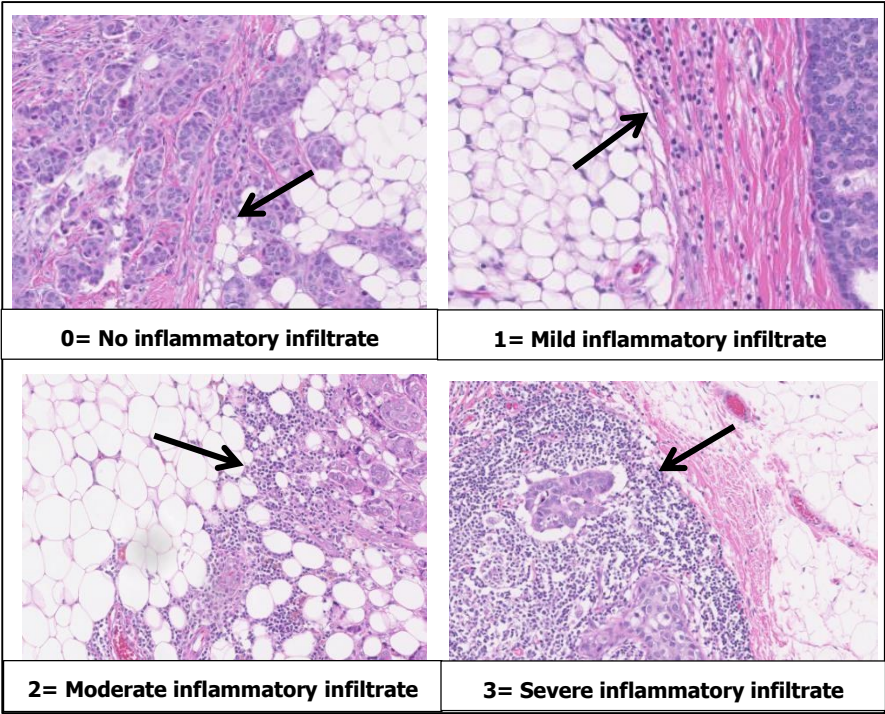

C)

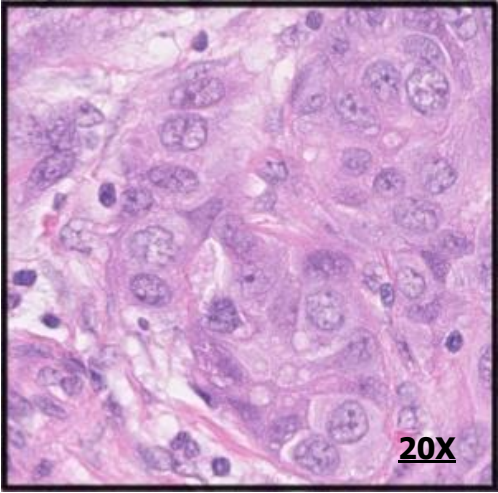

Supplement: Supplementary file 2 — Additional file 2: Figure S1. Estimation of inflammation reaction methods. (A) Delimitation of tumor area and estimation of tumor cell percentage (TCP) and tumor stroma percentage (TSP); (B) Scoring of general inflammatory infiltrate at the invasive margin (Klintrup criteria); (C) Representation of inflammatory cell counting at 20× magnification in one random box in the breast tumor (0.018 mm2). [file 12935_2018_663_MOESM2_ESM.pdf]

**Figure S2**

**A)**

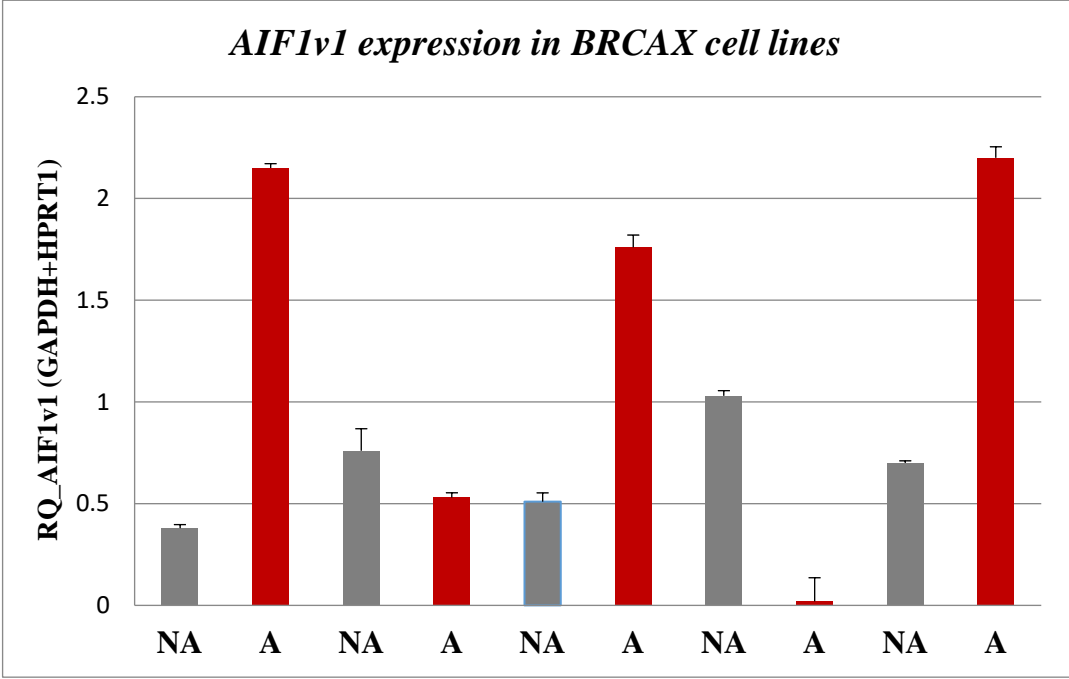

**B)**

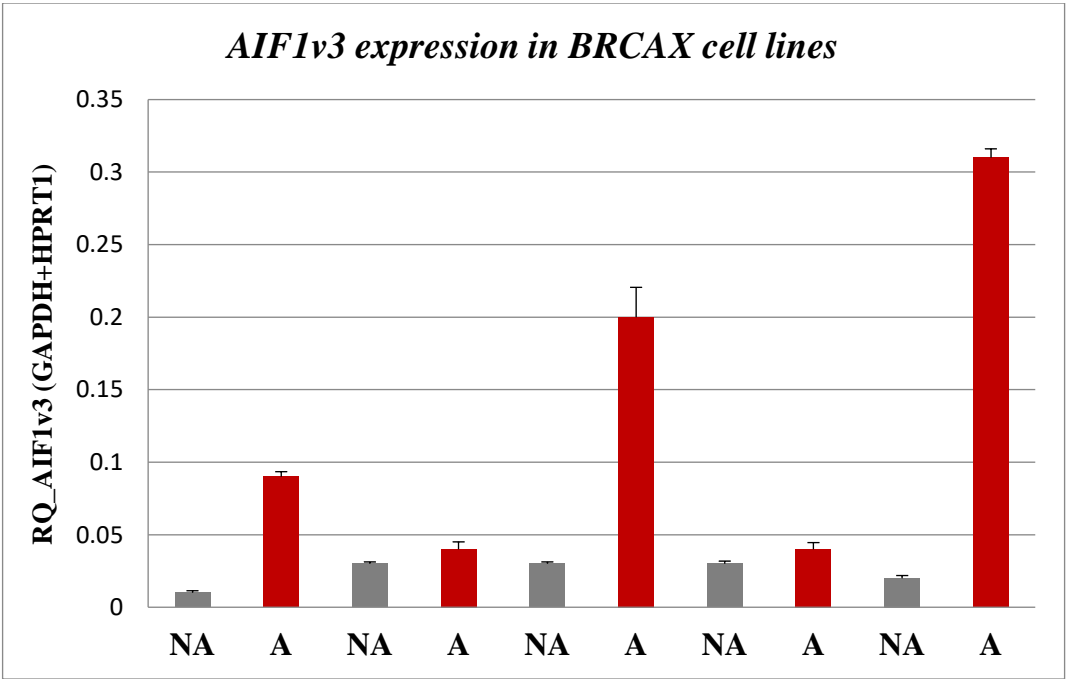

Supplement: Supplementary file 4 — Additional file 4: Figure S2. Validation of AIF1 expression in BRCAX immortalized lymphoblastoid cells (LCLs) by qRT-PCR in (A) affected sister and (B) non-affected sister. A = affected; NA = non-affected. [file 12935_2018_663_MOESM4_ESM.pdf]

Figure S3

A)

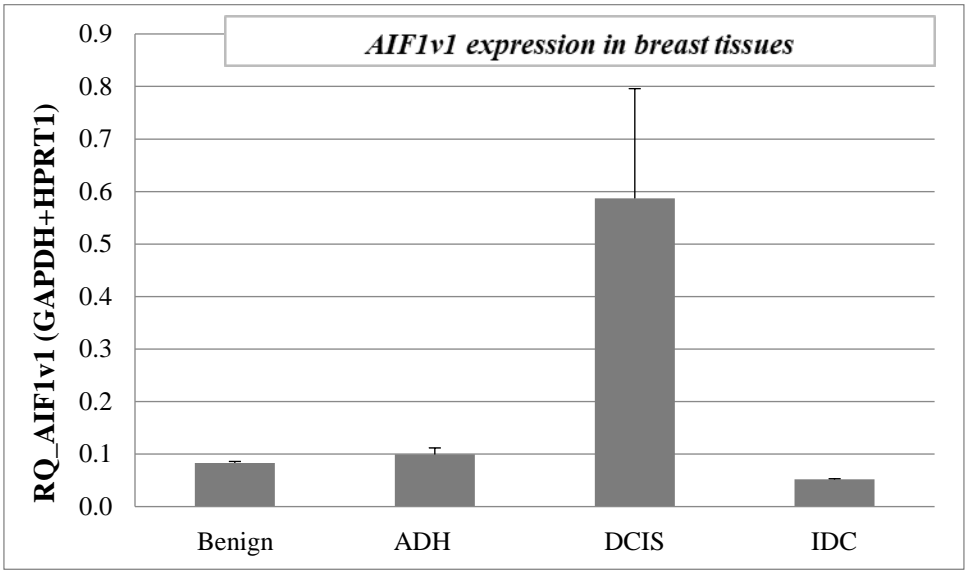

B)

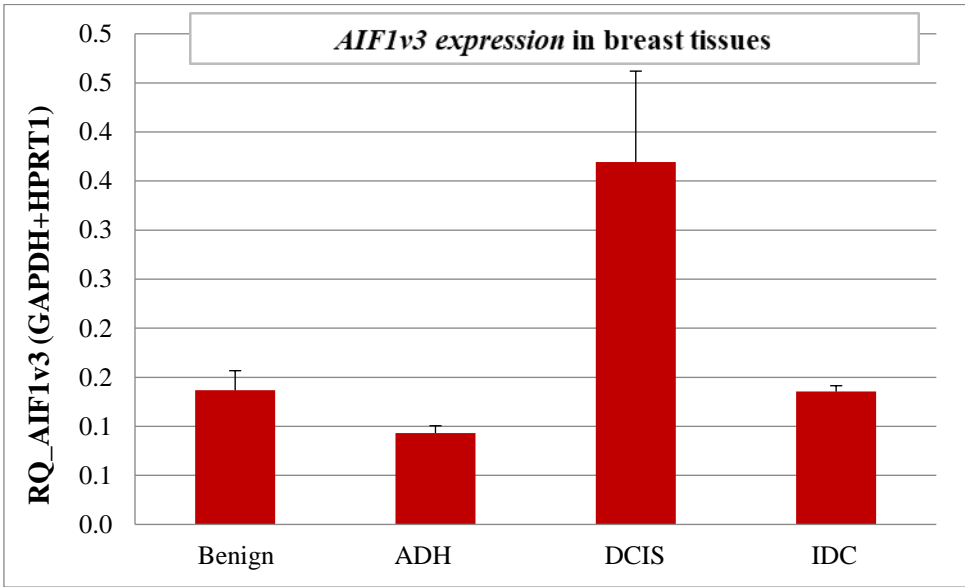

Supplement: Supplementary file 5 — Additional file 5: Figure S3. Expression of AIF1 in mammary tissue in isoforms (A) AIF1v1 and (B) AIF1v3. ADH = Atypical ductal hyperplasia; DCIS = Ductal carcinoma in situ; IDC = Invasive ductal carcinoma. [file 12935_2018_663_MOESM5_ESM.pdf]

Figure S4

A

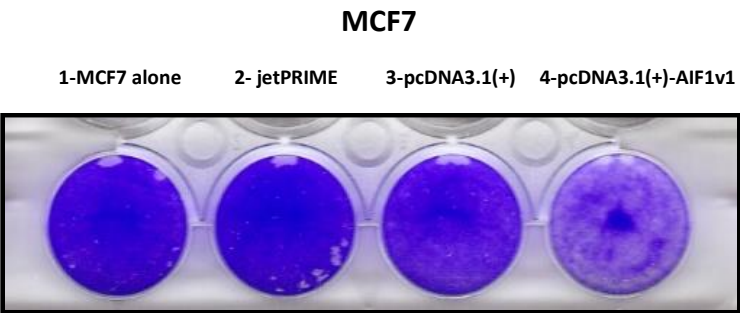

B

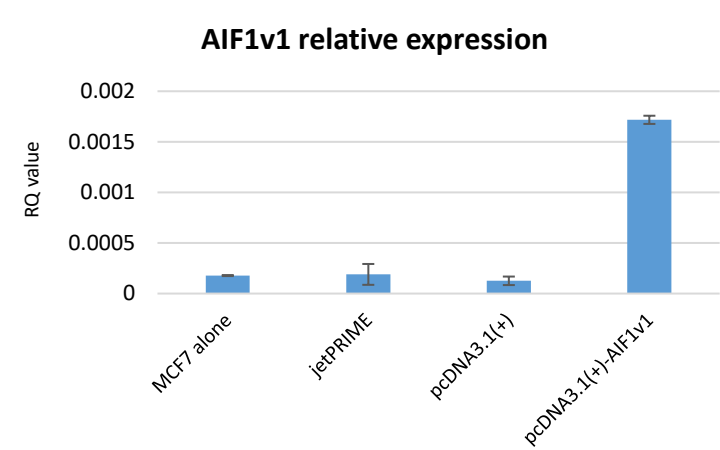

Supplement: Supplementary file 6 — Additional file 6: Figure S4. (A) Analysis of cell viability by crystal violet staining was performed on equal numbers of MCF7 breast cancer cells plated in a 12-well cell culture dish. The cells were transfected with (1) MCF7 alone (2) transfection agent (jetPRIME) 3) empty vector (pcDNA3.1 (+)) and (4) pcDNA3.1 (+)-AIF1v1 and let grown for 4 days. The purple color reflects the number of colonies formed after 4 days. A decrease in the number of colonies indicates decreased proliferation or increased cell death in presence of AIF1. (B) Relative expression levels of AIF1v1 mRNA by real-time PCR. The MCF7 cells seeded in 12-well plates were transfected with (1) MCF7 alone (2) transfection agent (jetPRIME) (3) empty vector (pcDNA3.1(+)) and (4) pcDNA3.1(+)-AIF1v1. HPRT1 was used as an internal control. [file 12935_2018_663_MOESM6_ESM.pdf]

Figure S5

A)

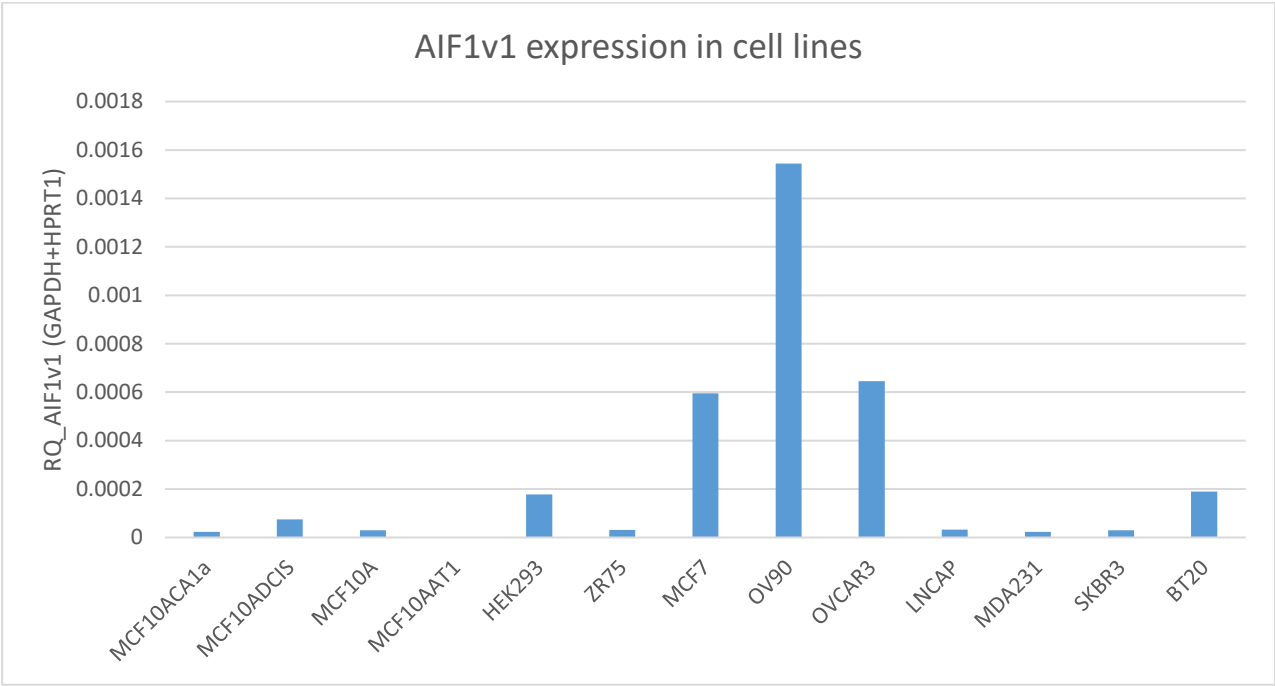

B)

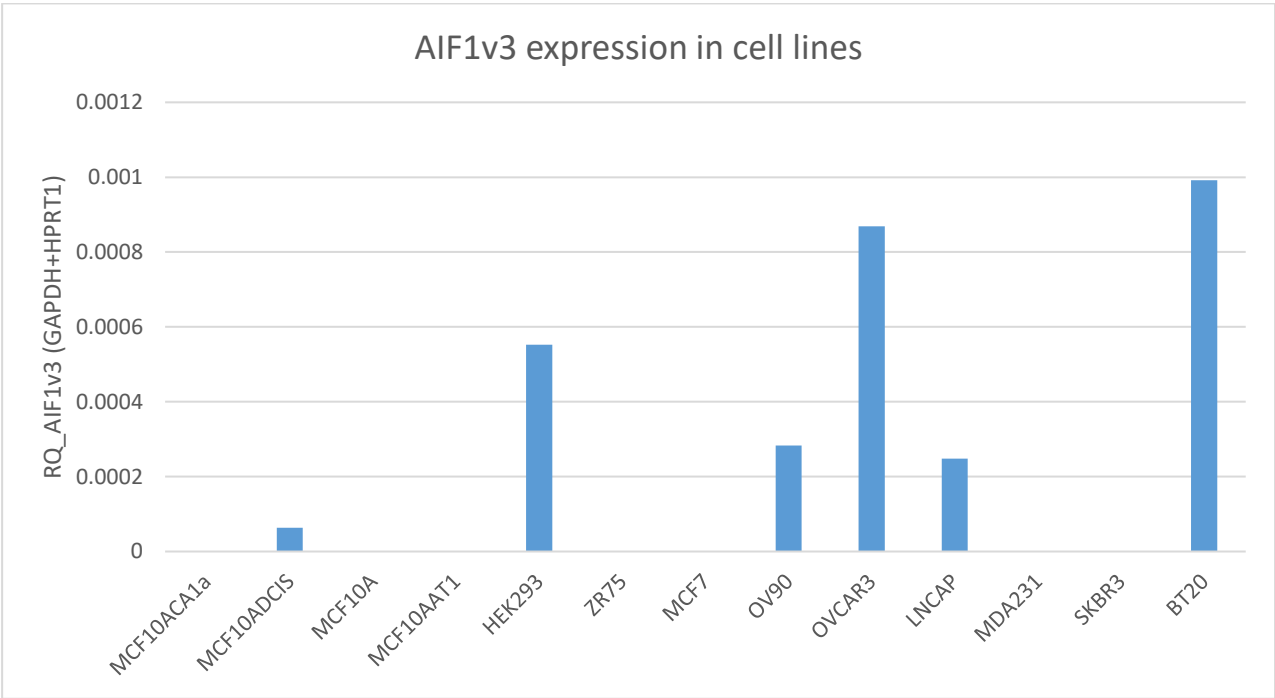

Supplement: Supplementary file 7 — Additional file 7: Figure S5. Expression of AIF1v1 (A) and AIF1v3 (B) in cancer cell lines. [file 12935_2018_663_MOESM7_ESM.pdf]

Figure S6

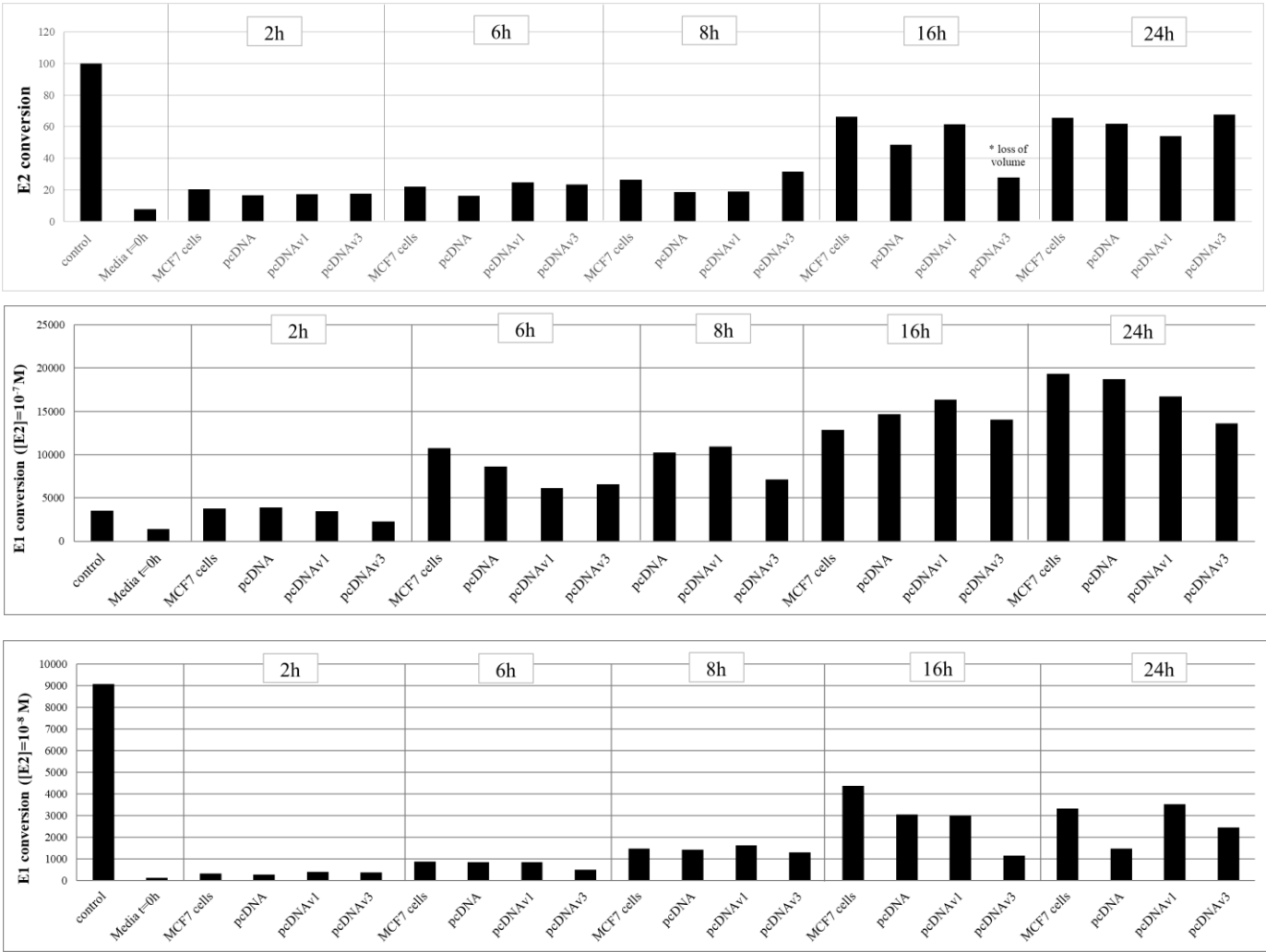

Supplement: Supplementary file 8 — Additional file 8: Figure S6. Conversion of E1/E2 at different incubation time periods. E1 = Estrone; E2 = 14C-estradiol. [file 12935_2018_663_MOESM8_ESM.pdf]

Figure S7

A)

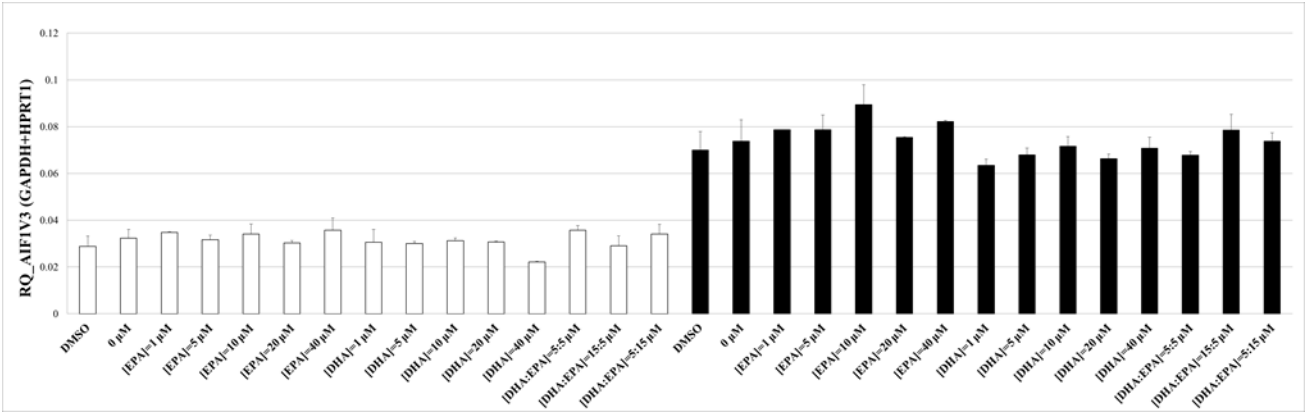

B)

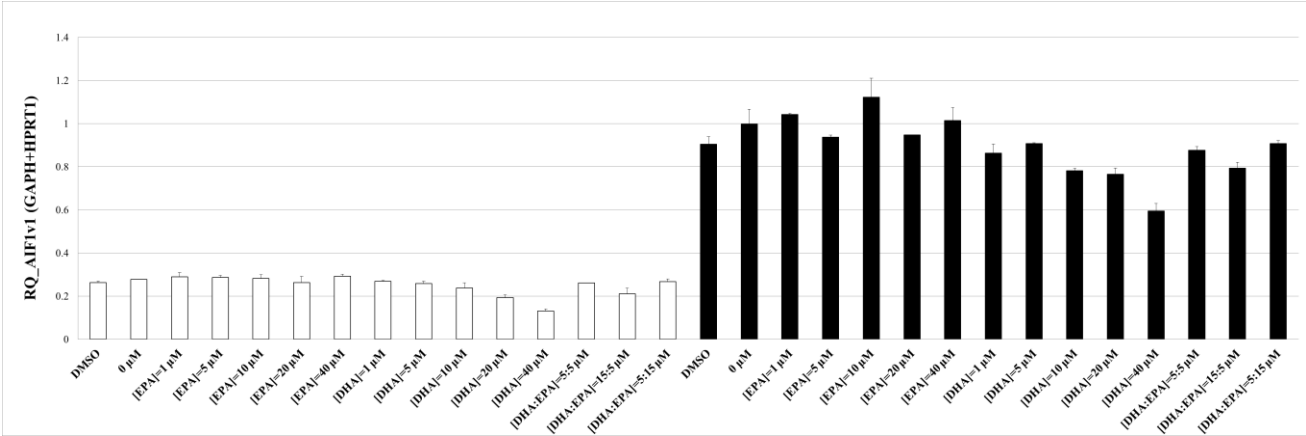

Supplement: Supplementary file 9 — Additional file 9: Figure S7. AIF1v1 (A) and AIF1v3 (B) expression at varying concentrations of EPA/DHA EPA = Eicosapentaenoic acid; DHA = Docosahexaenoic acid. [file 12935_2018_663_MOESM9_ESM.pdf]

Figure S8

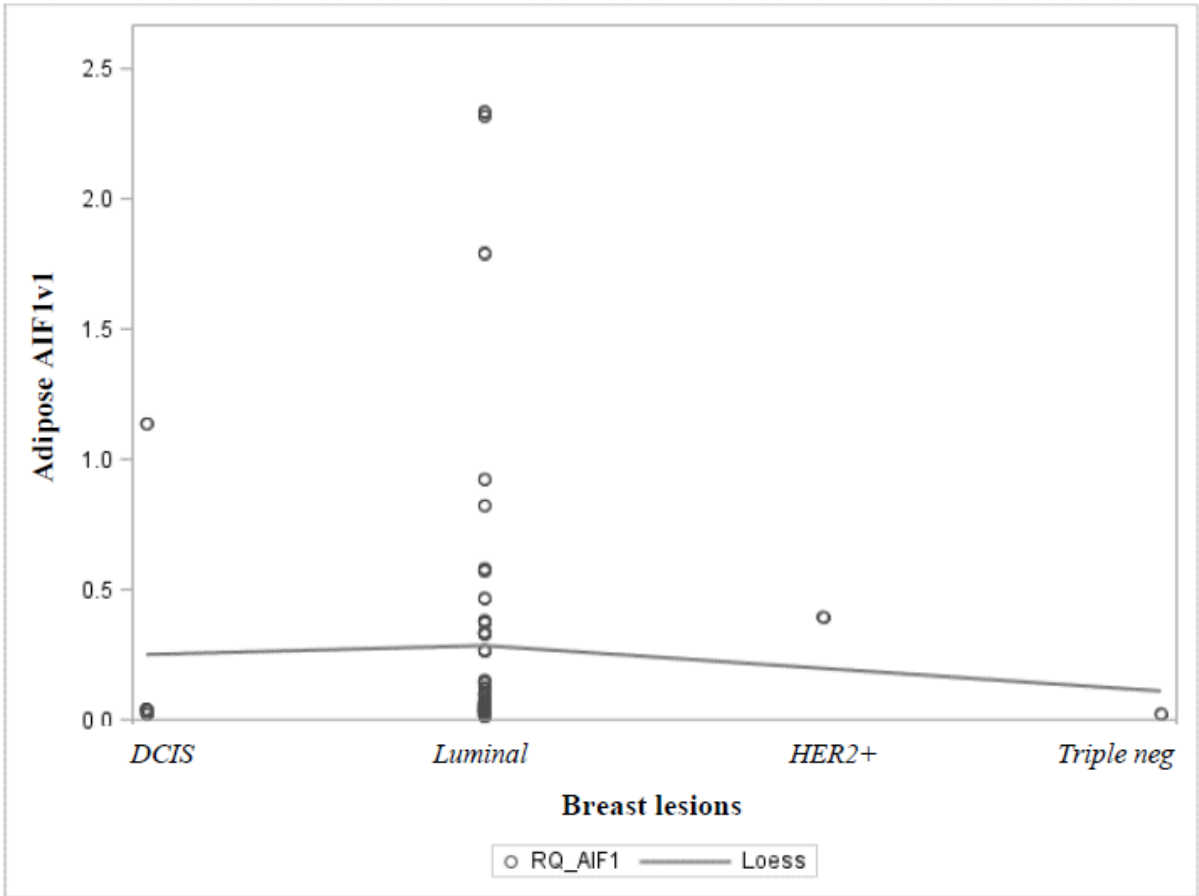

Supplement: Supplementary file 10 — Additional file 10: Figure S8. Distribution of breast adipose AIF1v1 expression in BC patients diagnosed with various breast tumors: ductal carcinoma in situ (DCIS), luminal A/B (ER+ and/or PR+), HER2+ (ER−/PR−/HER2+) and triple negative (ER−/PR−/HER2). [file 12935_2018_663_MOESM10_ESM.pdf]
