# Supplementary material for: An isoform of AIF1 involved in breast cancer
Source: Cancer Cell Int. 2018 Oct 22;18:167. doi: 10.1186/s12935-018-0663-3 (PMC6198497; doi:10.1186/s12935-018-0663-3)
Supplement: Supplementary file 3 — Additional file 3. Additional methods. [file 12935_2018_663_MOESM3_ESM.docx]

**An isoform of AIF1 involved in breast cancer**

Ferial Amira Slim, Geneviève Ouellette, Kaoutar Ennour-Idrissi, Simon Jacob, Caroline Diorio, Francine Durocher

**Supplementary Methods**

**1. MCF7 transfection and E1/E2 treatment**

The human breast cancer cell lines MCF7 were grown in Dulbecco’s modified Eagle’s medium with 10% fetal bovine serum (FBS) and 1% penicillin/streptomycin. cDNAs for AIF1v1 and AIF1v3 were cloned into the pcDNA3.1(+) vector by T4 DNA ligase. Primer sequences for human AIF1v1 and AIF1v3 are described in Additional file 1: Table S1. AIF1v1-pcDNA3.1 (pcDNAv1), AIF1v3-pcDNA3.1 (pcDNAv3), and empty vector pcDNA3.1 (pcDNA) were transfected, respectively, into MCF7 by JetPrime transfection reagent (Polyplus) according to the manufacturer’s protocol. Vector alone was used as a negative control. Equal numbers of stable transfectants were seeded into 24-well plates at a density of 150 × 10³ cells per well.

After transfection, cells were incubated for 24 hours and then treated with various concentrations of 14C-estrone (14C-E1; d1:100) or 14C-17β-estradiol (14C-E2; 10-7M, 10-8M, 10-9M or 10-10M) followed by 0,2,6,8,16 and 24 hours of incubation at 37°C under a 5% CO2 atmosphere. Media were then collected for steroid extraction and 1D-TLC.

**2. Steroid extraction and 1D Thin Layer Chromatography measurements**

Steroids from transfected MCF7 culture media were extracted twice with 2 ml diethyl ether followed by drying under nitrogen gas. Dichloromethane was used to dissolve the steroids which were applied to silica gel thin layer chromatography (TLC) plates. Plates were developed in a sealed glass chamber containing 100 ml of toluene/acetone (4:1) and quantified by phosphor autoradiography. Image J software was used for the calculations of estrone/estradiol conversion rate (NIH, Bethesda, MD, USA).

**3. Crystal Violet Assay for Determining Viability of Cultured Cells**

Crystal violet staining was performed 4 days post MCF7 transient transfection (jetPRIME, polyplus transfection) in 12-well plates. Plates were washed gently with PBS 1X, and 500ul of staining solution (0.4% crystal violet and 20% methanol) were added per well. After 15 minutes at room temperature, the plates were washed with running tap water.
